# The CECR2 bromodomain displays distinct binding modes to select for acetylated histone proteins versus non-histone ligands

**DOI:** 10.1101/2024.12.09.627393

**Published:** 2024-12-11

**Authors:** Margaret Phillips, Elizabeth D. Cook, Matthew R. Marunde, Marco Tonelli, Laiba Khan, Amy Henrickson, James M. Lignos, Janet L. Stein, Gary S. Stein, Seth Frietze, Borries Demeler, Karen C. Glass

**Affiliations:** 1 Department of Pharmacology, Larner College of Medicine, University of Vermont, Burlington, VT, 05405, USA; 2 EpiCypher Inc., Durham, North Carolina 27709, USA; 3 National Magnetic Resonance Facility at Madison and Department of Biochemistry, University of Wisconsin-Madison, Madison, WI, 53706, USA; 4 Department of Chemistry and Biochemistry, University of Lethbridge, Lethbridge, AB T1K 3M4, Canada; 5 Department of Biochemistry, Larner College of Medicine, University of Vermont, Burlington, VT, 05405, USA; 6 Department of Biomedical and Health Sciences, University of Vermont, Burlington, VT 05405, USA.; 7 University of Vermont Cancer Center, Burlington, VT 05405, USA.

## Abstract

The cat eye syndrome chromosome region candidate 2 (CECR2) protein is an epigenetic regulator involved in chromatin remodeling and transcriptional control. The CECR2 bromodomain (CECR2-BRD) plays a pivotal role in directing the activity of CECR2 through its capacity to recognize and bind acetylated lysine residues on histone proteins. This study elucidates the binding specificity and structural mechanisms of CECR2-BRD interactions with both histone and non-histone ligands, employing techniques such as isothermal titration calorimetry (ITC), nuclear magnetic resonance (NMR) spectroscopy, and a high-throughput peptide assay. The CECR2-BRD selectively binds acetylated histone H3 and H4 ligands, exhibiting a preference for multi-acetylated over mono-acetylated targets. The highest affinity was observed for tetra-acetylated histone H4. Neighboring post-translational modifications, including methylation and phosphorylation, modulate acetyllysine recognition, with significant effects observed for histone H3 ligands.

Additionally, this study explored the interaction of the CECR2-BRD with the acetylated RelA subunit of NF-κB, a pivotal transcription factor in inflammatory signaling. Dysregulated NF-κB signaling is implicated in numerous pathologies, including cancer progression, with acetylation of RelA at lysine 310 (K310ac) being critical for its transcriptional activity. Recent evidence linking the CECR2-BRD to RelA suggests it plays a role in inflammatory and metastatic pathways, underscoring the need to understand the molecular basis of this interaction. We found the CECR2-BRD binds to acetylated RelA with micromolar affinity, and uses a distinctive binding mode to recognize this non-histone ligand. These results provide new insight on the role of CECR2 in regulating NF-κB-mediated inflammatory pathways.

Functional mutagenesis of critical residues, such as Asn514 and Asp464, highlight their roles in ligand specificity and binding dynamics. Notably, the CECR2-BRD remained monomeric in solution and exhibited differential conformational responses upon ligand binding, suggesting adaptive recognition mechanisms. Furthermore, the CECR2-BRD exclusively interacts with nucleosome substrates containing multi-acetylated histones, emphasizing its role in transcriptional activation within euchromatic regions. These findings position the CECR2-BRD as a key chromatin reader and a promising therapeutic target for modulating transcriptional and inflammatory processes, particularly through the development of selective bromodomain inhibitors.

## INTRODUCTION:

CECR2 is a regulatory subunit of ATP-dependent chromatin remodeling complex CERF (CECR2 containing remodeling factor)^1^. Together with ISWI ATPases, SNF2L (SMARCA1) and SNF2H (SMARCA5), CECR2 regulates nucleosome organization to facilitate DNA accessibility during neurulation, spermatogenesis, and other developmental processes^1, 2^. These chromatin remodeling complexes also play critical roles in transcription and replication by modulating nucleosome spacing^3, 4^. Additionally, CECR2 contributes to the DNA damage response (DDR) through its bromodomain, which inhibits γ-H2AX activity^5^.

Inflammation plays an essential role in the initiation and progression of cancer^6^. The nuclear transcription factor-kappa B (NF- κB) is a crucial mediator of inflammatory responses in carcinogenesis^7^. The most abundant, activated form of NF-κB, is composed of a heterodimer of the p65 (RelA) and p50 (RelB) subunits^8^. Acetylation of lysine at residue 310 in RelA is required for optimal transcriptional activity of the NF-κB complex^9^. A recent study identified that CECR2 interacts with the acetylated K310 in RelA, which enhanced NF-κB signaling and increased the expression of pro-inflammatory and pro-metastatic factors^10^. Moreover, inhibition of CECR2 bromodomain (CECR2-BRD) binding activity reduced the expression of NF-κB mediated immune response and pro-metastatic genes^10^. CECR2 has also been recently identified as a potential prognostic marker for patients with glioma^11^. Thus, targeting CECR2 and its bromodomain activity represents a promising target for modulating NF-κB signaling and associated inflammation in cancer.

The full-length CECR2 is a multidomain protein that contains an N-terminus DDT domain (DNA-binding homeobox and different transcription factors), an AT-hook, and a bromodomain^4, 12^. The DDT and the AT-hook domains have been shown to bind DNA^13, 14^, whereas the bromodomain recognizes acetylated lysine^15^. Although the CECR2-BRD has been studied for its ability to recognize acetylated histones, a comprehensive understanding of how it ‘reads’ acetyllysine is still lacking. Moreover, limited information exists on how recognition of these histone modifications influences the chromatin remodeling activity of CECR2.

Over the past decade, research has demonstrated that bromodomains are excellent drug targets due to their crucial role in gene regulation, chromatin remodeling, and protein-protein interactions^16^. Understanding CECR2-BRDs role in these processes would provide valuable insights for the development of selective inhibitors to modulate its activity. In addition, outlining the molecular ligands of the CECR2 bromodomain as a part of the chromatin remodeler CERF would provide insight into its function in chromatin dynamics. To characterize the molecular mechanisms of the selective recognition of acetylated lysine by CECR2 bromodomain, we employed a combination of techniques including peptide array, isothermal titration calorimetry (ITC), and Nuclear Magnetic Resonance (NMR) spectroscopy. Our findings reveal a unique binding mode of the CECR2 bromodomain, offering new perspectives on its ability to selectively recognize acetyllysine modifications on both histone and non-histone proteins.

## METHODS:

### Plasmid construction

A construct encoding amino acids 424–538 of the human CECR2-BRD-containing protein (UniProt code: Q9BXF3–1) was codon optimized for expression in *Escherichia coli*, and cloned into a pGEX-6P-1 plasmid containing an N-terminal GST tag and a PreScission Protease cleavage site (GenScript, Piscataway, NJ, USA). The plasmid was transformed into *E. coli* BL21(DE3) pLysS competent cells (Novagen, MA, USA) for protein expression.

### Protein expression and purification

The GST-CECR2-BRD-containing BL21(DE3) pLysS cells were cultivated following established protocols^17, 18^. Briefly, cells were grown in 2 L of Terrific broth (TB) media, or ^15^N ammonium chloride and ^13^C-glucose-supplemented minimal media, with ampicillin and chloramphenicol antibiotics added, at 37°C. Once the optical density at 600 nm (O.D. 600) reached 0.6, the temperature was reduced to 20°C. Subsequently, the cells were grown until the O.D. 600 reached a range of 0.8–1.0 and then induced with 0.1 mM isopropyl β-D-1-thiogalactopyranoside (IPTG). The induced cells were further grown overnight at 20°C. Following induction, the cells were harvested by centrifugation at 5,000 RPM for 10 minutes at 4°C. The resulting cell pellet was resuspended in 100 mL of lysis buffer, which contained 50 mM Tris–HCl pH 7.5, 500 mM NaCl, 5% glycerol, 0.05% Nonidet P-40 alternative, 1 mM dithiothreitol (DTT) and supplemented with 0.1 mg/mL of lysozyme and 1 tablet of protease inhibitor (Pierce protease inhibitor tablets, EDTA-free, ThermoFisher). The cells were then lysed using sonication, and the resulting cell lysate was centrifuged at 10,000 RPM for 20 minutes. The supernatant was mixed with glutathione agarose resin (ThermoFisher) and incubated at 4°C with gentle agitation for 1 hour. Afterward, the suspension was transferred to Econo-Column Chromatography Columns (Bio-Rad) and washed with wash buffer (20 mM Tris–HCl pH 7.5, 500 mM NaCl, 5% glycerol, and 1 mM DTT). The GST tag was then cleaved by incubating the washed beads in wash buffer supplemented with PreScission Protease (GE Healthcare) overnight at 4°C. The eluted CECR2-BRD protein was subsequently concentrated to a total volume of approximately 3 mL. For dCypher assays, the GST-tag was retained by eluting with 50 mM reduced glutathione and free glutathione removed via dialysis (48 hours) into 20 mM Tris-HCl pH 8.0, 200 mM NaCl, 1 mM DTT, and 20% glycerol. The protein concentration was determined by measuring the protein absorbance at 280 nm. The purity of the CECR2-BRD was confirmed through sodium dodecyl sulfate-polyacrylamide gel electrophoresis (SDS-PAGE) stained with GelCode Blue Safe protein stain (ThermoFisher).

### dCypher Binding Assays

The approach was performed as previously described with minor modifications^18, 19^. In brief, 5 μL GST-CECR2-BRD was combined with 5 μL histone peptides (100 nM) or PTM-defined nucleosomes (10 nM) and incubated 30 minutes in a 384-well plate. Binding was detected by adding a 10 μL mixture of 2.5 μg/mL AlphaLISA glutathione Acceptor (Revvity AL109M) and 10 μg/mL streptavidin Donor beads (Revvity 6760002). After a 60-minute incubation, Alpha counts were measured on a PerkinElmer 2104 Envision plate reader (680 nm laser excitation, 570 nm emission filter ± 50 nm bandwidth). Reagents for peptide assays were prepared in: 50 mM Tris pH 7.5, 50 mM NaCl, 0.01% Tween-20, 0.01% BSA, 1 mM TCEP, 0.0004% Poly-L-Lysine. For nucleosomes they were prepared in: 20 mM Tris pH 7.5, 250 mM NaCl, 0.01% NP-40, 0.01% BSA, 1 mM DTT. Binding curves were plotted in GraphPad Prism 10 using 4-parameter logistic nonlinear regression curves. Relative EC_50_ (EC_50_^rel^) values were computed in GraphPad Prism; the concentration of GST-CECR2-BRD necessary to elicit 50% of maximal binding signal. Assay points exceeding the hook point were excluded from EC_50_^rel^ calculations^19, 20^. All binding interactions were performed in duplicate, and incubations performed at room temperature under subdued lighting.

### Peptide synthesis

All modified and unmodified peptides were obtained from GenScript (Piscataway, NJ, USA). All histone H4 peptides (1–24) were synthesized with N-terminal acetylation (Nα-ac) and C-terminal amidation, while all H3 peptides (1–24) contain only a C-terminus amidation. The RelA peptides (305–315) contain no N-term acetylation or C-term amidation. All peptides were purified with HPLC to reach 98% purity and confirmed using Mass Spectrometry analysis. The amino acid sequences of each peptide can be found in Table 1 and Table 3.

### Isothermal titration calorimetry

The CECR2-BRD was dialyzed for 48 hours into 20 mM sodium phosphate buffer, pH 7.0, 150 mM NaCl, and 1 mM tris(2-carboxyethyl) phosphine (TCEP) (ThermoFisher). All ITC experiments were performed at 5°C using a MicroCal PEAQ-ITC instrument (Malvern Analytical). All ligands were loaded into the syringe at concentrations ranging from 0.6 mM to 2.5 mM, while the CECR2-BRD was present in the sample cell at concentrations ranging from 0.05 mM to 0.10 mM. The histone ligand was injected into the sample cell in a series of 20 individual, 2 μL injections, with intervals of 150 s and continuous stirring at 750 RPM. Prior to these injections, a 0.4 μL preliminary injection of the peptide ligand was made but excluded from data integration and binding constant calculation. Control runs were performed by injecting histone peptide into the protein buffer under the same conditions, and the resulting heat of dilution was subtracted from the ligand-protein runs to correct for this effect. Data analysis was conducted using MicroCal PEAQ-ITC software. A single set of binding site models was applied to fit the data and determine the stoichiometry (N) and binding constant (*K*_*D*_). Binding experiments were performed in triplicate, while non-binding runs were conducted in duplicate. The mean *K*_D_ value was calculated from the average of the three binding runs, and the standard deviation was determined based on the mean. All *K*_D_ values and their corresponding stoichiometries are reported in Table 1 and Table 3.

### Analytical Ultracentrifugation

To determine the interaction potential between the CECR2-BRD and mono- and di-acylated histone peptide ligands, and their effect on CECR2-BRD oligomerization, we analyzed mixtures between the two molecules by sedimentation velocity (SV) at various molar ratios including:

1. CECR2-BRD (apo), measured at 233 nm
2. CECR2-BRD + H3K4acK9acK14acK18ac at a 1:2 molar ratio, measured at 236 nm
3. CECR2-BRD + H3K4acK9acK14acK18ac at a 1:2 molar ratio, measured at 232 nm
4. CECR2-BRD + H3K4acK9acK14acK18ac at a 1:0.5 molar ratio, measured at 238 nm
5. CECR2-BRD + H3K14ac at a 1:0.5 molar ratio, measured at 239 nm
6. CECR2-BRD + H4K5acK8acK12acK16ac at a 1:0.5 molar ratio, measured at 232 nm
7. CECR2-BRD + H4K5acK8acK12acK16ac at a 1:1 molar ratio, measured at 235 nm
8. CECR2-BRD + H4K8ac at a 1:0.5 molar ratio, measured at 239 nm

The CECR2-BRD protein concentration ranged from 5.2–25.1 μM, multiple molar ratios were used in order to ensure the ligand concentrations would be above the K_D_ of the protein:ligand complex as determined by ITC (Table 1).

All samples were measured in UV-intensity mode, and to optimize signal-to-noise ratio and maintain all signals within the dynamic range of the absorbance detector, the wavelength of data collection was optimized for optical densities between 0.5–0.8 OD determined by UV spectroscopy using a ThermoFisher Genesys 10s benchtop spectrophotometer prior to AUC analysis. All samples were measured in a buffer containing 150 mM NaCl, 20 mM NaPO4, adjusted to pH 7.0, with 1 mM TCEP added to prevent cysteine oxidation. The experiment was performed at 35,000 rpm for 15 hours at 5°C. Intensity data were collected in an Optima AUC at the Canadian Center for Hydrodynamics at the University of Lethbridge. All data were processed with UltraScan^24^, as described previously^25^. Experimental data were fitted using the 2-dimensional spectrum analysis (2DSA)^26^, in a multi-step refinement process to eliminate time- and radially-invariant noise, and to define boundary conditions. The refined data were then further analyzed using the 2DSA iterative (IT) and 2DSA Monte Carlo (MC) methods^27^. To compare the sedimentation profiles of each sample, diffusion-corrected integral sedimentation coefficients were generated and overlayed using the van Holde-Weischet^28^.

### Nuclear Magnetic Resonance Spectroscopy

CECR2-BRD was expressed and purified for NMR experiments following previously established methods^17, 18^. Briefly, uniformly labeled ^15^N CECR2-BRD samples in buffer containing 20 mM Tris-HCl pH 6.8, 150 mM NaCl, 1 mM TCEP, and 10% D_2_O were purified at 0.30 mM for chemical-shift perturbation experiments.

All two-dimensional (2D) ^15^N-HSQC (heteronuclear single quantum interference) experiments were acquired at 25°C using a 600 MHz Bruker AVANCE III spectrometer equipped with a z-gradient 1.7 mm TCI probe at the National Magnetic Resonance Facility at Madison (NMRFAM). The experimental setup for all 2D experiments was kept similar to our previous studies^17, 18^. Histone and non-histone ligands were added in increasing concentrations of 1:0.5, 1:1, 1:2.5, 1:5, and 1:10 molar ratios of protein to the ligand, except Histone H4 K5acK8acK12acK16ac for which the highest molar concentration was kept at 1:5. Chemical Shift Perturbations (CSP) from the final titration point were calculated and normalized as described by Williamson^21^, using the equation:

$$\Delta \delta =\surd \Delta {\left(\delta_{H}\right)}^{2}+{\left(\alpha \cdot \Delta \delta_{N}\right)}^{2}$$

Where α=0.2 for glycine, and 0.14 for all other residues. $\Delta \delta_{H}$ and $\Delta \delta_{N}$ represent chemical shifts in ppm for hydrogen and nitrogen atoms, respectively. CSPs were plotted as a bar graph to identify CECR2-BRD residues perturbed in response to adding specific histone ligands. These were calculated using the standard deviation (Δ ppm/ Standard deviation ppm, where standard deviation was calculated for each histone ligand) of the Euclidean chemical change of the residues as described previously^17, 18^.

For the backbone assignments, ^15^N, ^13^C labeled CECR2-BRD was prepared at 0.5 mM concentration in 20 mM Tris–HCl, pH 6.8, 150 mM NaCl, 1 mM TCEP, and 10% D_2_O. 2D ^15^N-1H NHSQC, 3D HNCACB, and 3D CBCACONH experiments were recorded at 25°C on a 750 MHz Bruker AVANCE III spectrometer equipped with a z-gradient 1.7 mm TCI probe at the National Magnetic Resonance Facility at Madison (NMRFAM). All spectra were processed using NMRPipe and analyzed using NMRFAM-Sparky and POKY^22, 23^.

## RESULTS:

### CECR2 bromodomain broadly recognizes acetylated histone ligands and tolerates other combinatorial PTMs

**Figure 1.**
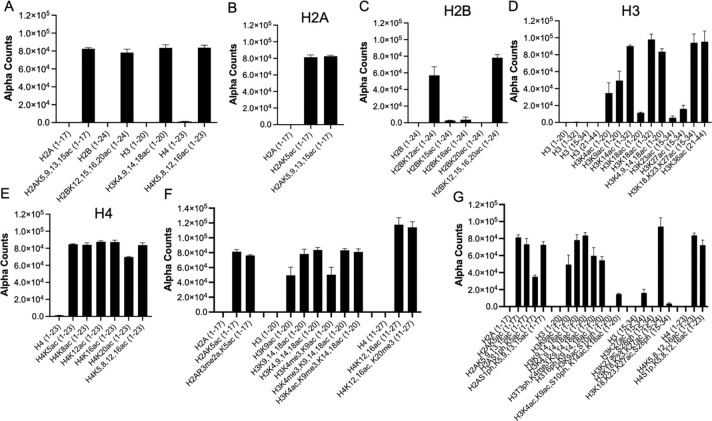
CECR2 bromodomain broadly recognizes acetylated histone peptides. Using the dCypher assay, a panel of 287 histone peptides was probed with 26 nM GST-CECR2-BRD to assess binding interactions. The X-axis represents the identity and amino acid coverage of each histone peptide, while the Y-axis measures the Alpha Counts indicating binding strength. A) CECR2 shows robust binding across multi-acetylated H2A, H2B, H3, and H4 histone peptides. B-E) Detailed binding profiles of CECR2 with single and multi-acetyl peptides of H2A, H2B, H3, and H4, respectively. F) CECR2 demonstrates tolerance for methylation on H2A, H3, and H4 peptides, maintaining binding strength in the presence of adjacent methylated residues. G) Phosphorylated residues negatively impact certain CECR2 binding to acetylated peptides. All values shown are the averages ± standard deviation (n = 2).

There is limited understanding of CECR2-BRD interactions with modified chromatin, thus we sought to investigate its binding to modified histone targets using the high-throughput dCypher approach. This method employs a no-wash bead-based assay platform, AlphaScreen, to assess interactions between the CECR2-BRD and PTM-defined histone ligands in 384-well plates^29^. By titrating the CECR2-BRD against selective peptides including, histone H4K5acK8acK12acK16ac residues 1–23 (H4Tetra^ac^) and unmodified H4 (1–23 aa), we observed a robust interaction to H4Tetra^ac^ (EC_50_^rel^: 7.2 nM) and determined an optimal probing concentration (26 nM; Supp. Figure S1). We then examined CECR2-BRD binding to a comprehensive panel of 287 histone peptides representing diverse sets of single and combinatorial PTMs (methylation, acylation, phosphorylation, etc.) across histones H2A, H2B, H3, and H4 (Supp. Table 1 for all data). Robust and specific binding was observed toward acetylated peptides with indistinguishable interactions for multi-acetylated peptides across H2A, H2B, H3, and H4 (Figure 1). Binding was also observed with singly acetylated targets, with the strongest interactions occurring for acetylated histone H3 and H4 peptides (Figures 1D–E). Additionally, we investigated the impact of neighboring PTMs in the form of methylation (Figure 1F) and phosphorylation (Figure 1G) on acetyllysine recognition. Methyl groups in close proximity had no effect on the recognition of the adjacent acetylation marks, while phosphorylation mostly impacted acetyl interactions on the histone H3 tail.

**Figure 2.**
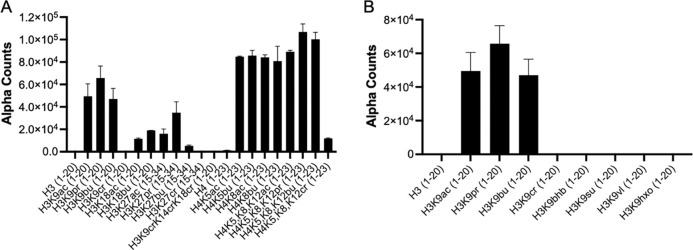
CECR2 bromodomain equally binds acetyl, propionyl, and butyryl. The binding of CECR2-BRD was examined using dCypher and acylated histone peptides on H3 and H4. A) CECR2 shows robust binding to acetyl, propionyl, and butyryl modifications at various lysine residues on the H3 and H4. B) Investigation of multiple acyl modifications at H3K9 reveals CECR2-BRD only binding acetyl (ac, propionyl (pr), and butyryl (bu) residues while no binding was detectable to crotonyl (cr), beta-hydroxybutyryl (bhb), succinyl (su), valeryl (vl), or hexanoyl (hxo). All values shown are the averages ± standard deviation (n = 2).

Previous studies have shown that CECR2-BRD distinguishes butyrylation and other less bulky acyl groups from crotonylation moeities^30^. Building on this, we expanded our analysis to include additional histone peptides and bulkier acyl modifications. Our data indicate that CECR2-BRD exhibits similar preference for acetyl, propionyl, and butyryl modified histones H3 and H4 peptides (Figure 2). However, CECR2-BRD histone interactions were dramatically weakened by all crotonyl peptides. Furthermore, bulkier acyl modifications such as beta-hydroxybutyryl, succinyl, pentanoyl, and hexanoyl modifications were incompatible with CECR2-BRD binding (Figure 2B). Collectively, these results demonstrate that the CECR2-BRD selects for short-chain acyl modifications.

### The CECR2 bromodomain prefers multi-acetylated histone tail ligands

Binding affinity studies offer quantitative information about the interaction between bromodomains and their ligands, enabling a better understanding of the molecular recognition and binding dynamics involved in bromodomain-mediated processes. Following our initial screening, which demonstrated CECR2-BRD’s preference for acetylated H3 and H4 histone peptides (Figure 1), we prioritized quantifying its binding affinity to these ligands. Using isothermal titration calorimetry (ITC), we identified the preferred acetylated H3/H4 ligands of the CECR2 bromodomain (Table 1, Supp. Figure S2 and S3). Among the mono-acetylated H3/H4 ligands examined, CECR2-BRD preferentially bound histone H4K8ac, with an affinity of *K*_D_ = 15.4 ± 1.9 μM. Among multi-acetylated H3/H4 ligands tested, the highest binding affinity was observed for the H4Tetra^ac^ ligand (*K*_D_ = 1.6 ± 0.2 μM). Conversely, H3Tetra^ac^ (H3K4acK9acK14acK18ac) bound with a slightly lower binding affinity (*K*_D_ = 28.0 ± 1.7 μM). To further evaluate the high binding affinity for the tetra-acetylated H4 ligand, we included various combinations of H4 acetylation marks and assessed their impact on CECR2-BRD binding (Table 1). Overall, our results indicate that the CECR2-BRD prefers multi-acetylated histones, especially histone H4. Interestingly, the residues surrounding the acetylated lysine on histone H3 peptides may influence binding affinity of the CECR2 bromodomain. The strongest binding affinity for mono-acetylated H3 peptides is observed when the adjacent amino acids are small nonpolar residues, such as glycine and alanine, as seen with H3K14ac. Whereas the binding affinity of the CECR2-BRD is weaker for the H3K4ac and H3K9ac ligands, which have adjacent residues that are larger and polar (Table 1).

### Specificity Determinants of the CECR2 Bromodomain Activity

**Figure 3:**
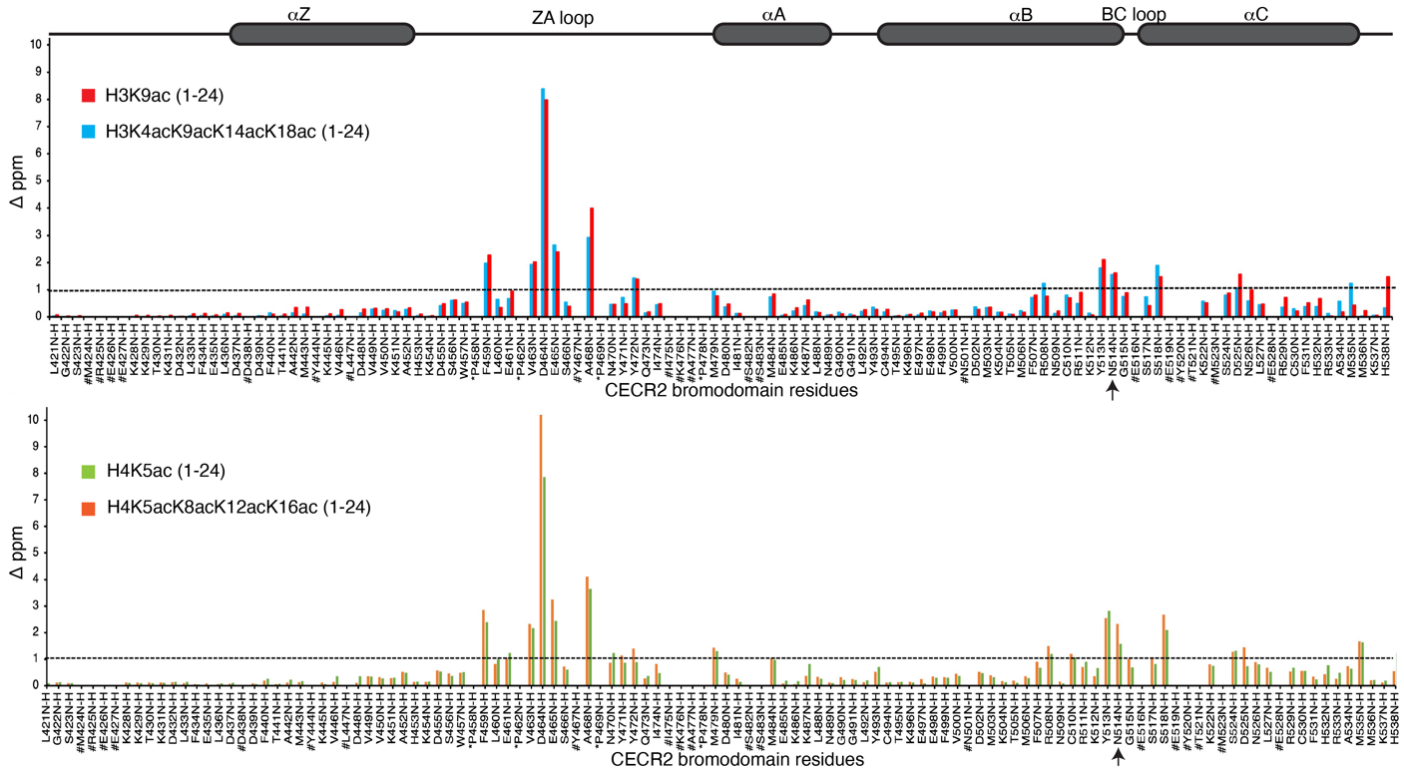
Normalized Chemical Shift Perturbation (CSP) plots for histone H3/H4 interaction with the CECR2 bromodomain. In the histogram plots above, the CECR2 bromodomain residues are along the x-axis, while their chemical shift (ppm) induced by adding histone ligands in a 1:10 (protein: peptide) molar ratio (except 1:5 for CECR2:H4K5acK8acK12acK16ac), along the y-axis. CSPs induced by different histone ligands are color-coded in the following order: red for H3K9ac, blue for H3K4acK9acK14acK18ac, green for H4K5ac, and orange for H4K5acK8acK12acK16ac ligand respectively. Prolines are marked by an asterisk (*), the conserved asparagine (N514) by an arrow, and the # symbol highlights unassigned residues. The dotted line represents the 1 standard deviation cut-off. The secondary structure containing four alpha-helices and ZA/BC loop regions of the CECR2 bromodomain are shown above the histogram.

Next, we sought to identify the molecular mechanisms involved in the recognition of these acetylated histones by the CECR2-BRD. Using NMR spectroscopy, we aimed to characterize the binding pocket of the CECR2-BRD. First, backbone residues of the CECR2-BRD were assigned using conventional three-dimensional (3D) NMR spectroscopy experiments (Supp. Figure S4). Next, to delineate the bromodomain-histone interaction site, 2D,^15^N-^1^H heteronuclear single quantum coherence (HSQC) NMR experiments were recorded in the absence and presence of increasing concentrations of the histone ligands (Figure 3, Supp. Figure S5–S8).

Consistent with previous studies of bromodomain-histone interactions, adding mono- or tetra-acetylated H3 and H4 peptides induced chemical shift perturbations (CSP) within a subset of CECR2-BRD residues. We observed significant CSPs in CECR2 residues lining the ZA and BC loops, and some residues within the αB and αC helices. The CECR2-BRD residues most perturbed upon histone ligand addition are the side-chain Hε-Nε of the W457 and backbone amide resonance of F459 from the ‘WPF’ shelf region. Backbone amide resonances of residues V463, D464, E465, and A468 from the ZA loop, along with Y513, conserved N514, and S518 from the BC loop were also significantly perturbed. A brief analysis of the CSPs highlight that CECR2-BRD employs similar or overlapping binding sites for binding the H3 and H4 histone ligands (Figure 3 top and bottom panels, and Supp. Figures S5–S8, respectively). Moreover, our titration data suggests that the CECR2-BRD recognizes acetylated histone H3 independently of the number of acetylation modifications. On the contrary multi-acetylated histone H4 displayed more intense CSPs when compared to the mono-acetylated ligand.

Given the high sensitivity of the residue D464 (ZA-loop of the CECR2-BRD) to the histone H3 and H4 interaction, we designed a D464A CECR2-BRD mutant to study the importance of this residue in CECR2-BRD interaction with acetylated H3 and H4 ligands. Since the conserved asparagine in bromodomains is also known to play a crucial role in bromodomain-histone interaction^31^ we created another mutant - N514A of the CECR2-BRD for comparative assessment. Together these mutations will shed light on the contributions of CECR2-BRD residues in coordinating histones.

In our ITC experiments, we exclusively utilized mono- and tetra-acetylated histone H3 and H4 ligands, which exhibited the highest affinity for the wild-type CECR2-BRD (Table 2, Supp. Figure S9 and S10). Our findings revealed that substituting residue N514 to Ala significantly reduced binding affinity to both mono-acetylated H3K14ac (*K*_D_ = 560.5 ± 54.4 μM) and H4K8ac (*K*_D_ = 518.7 ± 84.6 μM) ligands compared to the wild type (*K*_D_ = 34.2 ± 5.1 μM and *K*_D_ = 15.4 ± 1.9 μM, respectively).

Conversely, a D464 to Ala mutation led to only a modest decrease in the binding affinity of the H3K14ac ligand (*K*_D_ = 74.4 ± 3.1 μM), but a significant decrease in the binding affinity of the H4K8ac ligand (*K*_D_ = 239.0 ± 18.5 μM) compared to the wild type. Within the tetra-acetylated histone ligands, the N514A mutation had a more pronounced effect on the recognition of H4Tetra^ac^ ligands (*K*_D_ = 37.4 ± 5.4 μM) than H3Tetra^ac^ (*K*_D_ = 28.4 ± 3.4 μM) compared to the wild type (*K*_D_ = 1.5 ± 0.2 μM and 28.0 ± 1.7 μM, respectively). However, D464 to Ala mutation had lesser impact on either H3Tetra^ac^ (*K*_D_ = 63.3 ± 2.3 μM) or H4Tetra^ac (^*K*_D_ = 37.4 ± 5.4 μM) binding compared to the wild type.

### The CECR2 bromodomain is a functional monomer in solution

**Figure 4.**
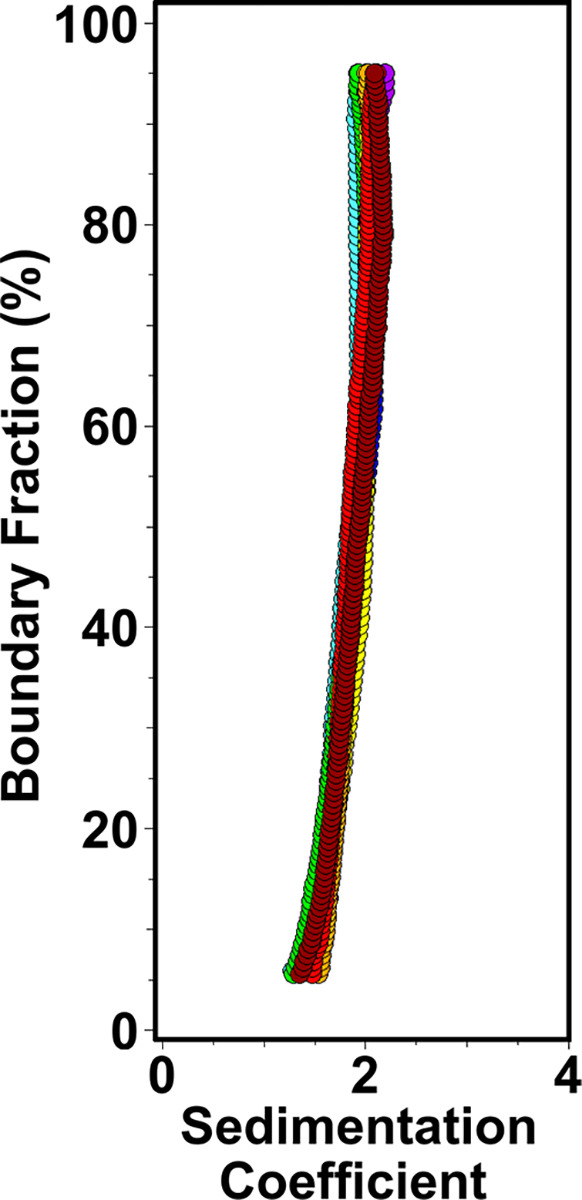
Sedimentation velocity analysis of the CECR2 bromodomain alone and in complex with acetylated histone H3 and H4 ligands. The sedimentation velocity distribution van Holde-Weischet overlay plot includes: CECR2-BRD apo control (purple), CECR2-BRD + H3K4acK9acK14acK18ac 1:2 (blue), CECR2-BRD + H3K4acK9acK14acK18ac 1:2 (cyan), CECR2-BRD + H3K4acK9acK14acK18ac 1:0.5 (orange), CECR2-BRD + H3K14ac 1:0.5 (red), CECR2-BRD + H4K5acK8acK12acK16ac 1:0.5 (green), CECR2-BRD + H4K5acK8acK12acK16ac 1:1 (yellow), CECR2-BRD + H4K8ac 1:0.5 (brown).

To determine if the CECR2-BRD exhibits self-association and oligomerization whilst interacting with histone peptide ligands, we performed analytical ultracentrifugation experiments. We tested stoichiometric mixtures of the CECR2-BRD with histone peptides containing single and multiple acetylation modifications including, H4K8ac, H3K14ac, H4K5acK8acK12acK16ac, and H3K4acK9acK14acK18ac. Sedimentation profiles for all CECR2-BRD and peptide mixtures are shown in Figure 4. All peptide mixtures with the CECR2-BRD sediment identically to the CECR2-BRD apo control, indicating that CECR2-BRD remains monomeric in the presence of histone peptide ligands. Shifts in the sedimentation profile were not observed upon addition of ligands to CECR2-BRD, therefore, binding of ligands to CECR2-BRD cannot be confirmed by AUC for any of the peptide or protein ratios examined. If any binding had taken place, the shift in sedimentation coefficient would have been very small, and likely below the resolution of this method in order to be detectable. Instead, we can conclude that CECR2 remains monomeric, because dimerization of the CECR2-BRD, which would be observable, is not detected.

### The CECR2 bromodomain interacts with acetylated RelA via a binding mode distinct from the histone recognition site

Acetylation of the transcription factor p65 (RelA), a member of the NF-κB family, modulates inflammatory responses and drives many of the pathophysiological functions of NF-κB^8, 9, 32, 33^. Specifically, acetylation of Lys310 of RelA is required to fully activate the transcriptional functions of NF-κB^9, 34^. Recently, CECR2 was identified as a critical epigenetic regulator that interacts with acetylated RelA, increasing the expression of NF-κB target pro-inflammatory genes and driving breast cancer metastasis^10^.

**Figure 5:**
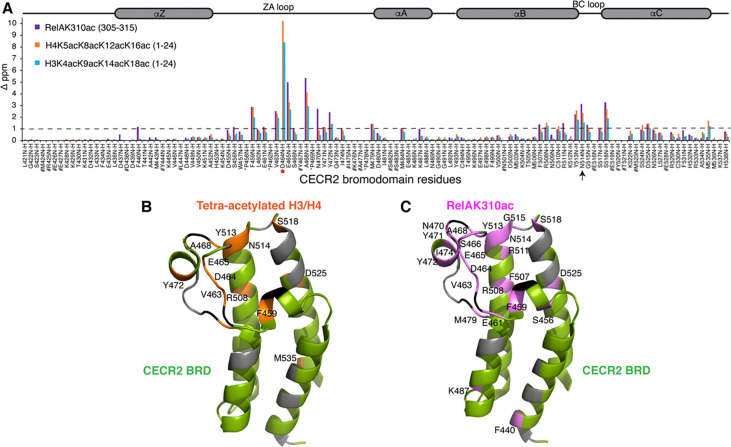
Comparing the Binding Pocket interactions of Acetylated RelA with Acetylated Histones. A) Normalized Chemical Shift Perturbation (CSP) plot for mono-acetylated RelA versus the tetra-acetylated histone H3/H4 ligands. CECR2-BRD residues are along the x-axis, while their chemical shift induced by adding various ligands is represented along the y-axis (D ppm). CSPs induced by mono-acetylated RelAK310ac (305–315) are shown in purple (1:10 molar ratio of CECR2: RelA), tetra-acetylated H4K5acK8acK12acK16ac (1–24) in orange (1:5 molar ratio of CECR2: H4), and H3K4acK9acK14acK18ac (1–24) in blue (1:10 molar ratio of CECR2: H3). Residue D464 was broadened beyond detection upon RelAK310ac addition and is highlighted in red asterisk. Prolines are marked by black asterisks (*), conserved asparagine N514 by an arrow, and the # symbol highlights unassigned residues. The dotted line represents the standard deviation cut-off of total CSPs. The secondary structure of the CECR2-BRD containing four alpha-helices and ZA/BC loop regions is shown above the histogram. B) Mapping the binding pocket of tetra acetylated H3 and H4 ligands onto the apo crystal structure of the CECR2-BRD (PDB ID: 3NXB). Highlighted in orange are the residues showing CSPs above standard deviation (>1 D ppm) upon ligand addition (1:5 for CECR2:H4K5acK8acK12acK16ac and 1:10 for CECR2:H3K4acK9acK14acK18ac molar ratio). Green residues are assigned, grey unassigned, while black residues denote Prolines. C) Mapping the binding pocket of acetylated RelAK310ac onto the apo crystal structure of the CECR2-BRD (PDB ID: 3NXB). Highlighted in pink are the residues showing CSPs above standard deviation (>1 D ppm) upon ligand addition (1:10 protein to peptide molar ratio).

We performed ITC with CECR2-BRD with RelA peptide (RelA residues 305–315) in the presence and absence of K310 acetylation and found that the BRD selects for acetylation mark with a binding affinity of *K*_D_ = 112.3 ± 4.2 μM (Table 3, Supp Figure S11). Next, to delineate the RelAK310ac binding pocket we performed 2D HSQC NMR titrations with ^15^N-labeled CECR2-BRD in the presence of increasing concentrations of the acetylated RelA peptide (Figure 5A). Overall, the CSP pattern for RelAK310ac interaction with CECR2-BRD was similar to the H3 or H4 interaction. However, there were some residues within the ZA and BC loop regions of the CECR2-BRD (E465, A468, N470, and Y472, including the conserved N514) that were more significantly perturbed upon RelA binding than either of the H3 or H4 ligands (Figure 5, Supp. Figure S12). Since the loops are mostly unstructured, more CSPs in these regions could indicate structural changes or alteration in the chemical environment of these residues upon ligand binding. Additionally, the conserved N514 plays a key role in coordinating acetylated RelA since mutation of N514 to Ala resulted in abolishing RelAK310ac binding (Table 3, Supp. Figure S10E). Of note is residue D464 which experienced the most significant ligand-induced signal broadening upon addition of higher concentrations of RelAK310ac ligand. Due to this, mapping the chemical shift perturbation of this residue was not possible (red asterisk in Figure 5A). Similarly, mutation of D464 to Alanine significantly reduced the binding affinity of CECR2-BRD to acetylated RelA peptide (Table 3, Supp. Figure S9E) further confirming its role in RelA interaction. CSP-guided mapping onto the apo crystal structure of the CECR2-BRD (PDB ID: 3NXB) delineates the binding pocket for the acetylated RelA ligand which is similar to the binding pocket of H3 or H4 ligands (Figure 5B and C). Overall, a comparison of chemical shift perturbations induced by acetylated RelA with the acetylated histone H3 and H4 ligands indicates that the interaction of the CECR2-BRD with acetylated RelA is similar to the coordination of the acetyllysine on histones. However, acetylated RelA binding involved more residues within the ZA loop as seen in Figure 5B and 5C. The differences in chemical shift perturbations also suggest that the CECR2-BRD may adopt a unique conformation or binding mode when interacting with acetylated RelA compared to the H3 and H4 histone ligands, potentially reflecting specific molecular recognition mechanisms or functional consequences related to its interaction with RelA.

**Figure 6:**
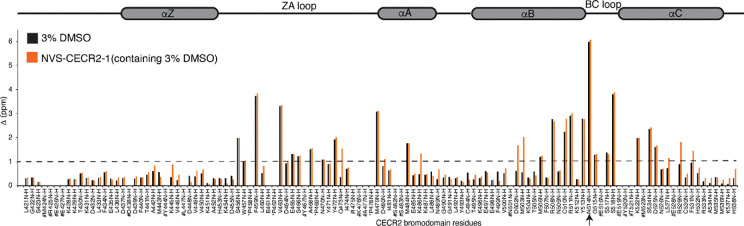
Normalized Chemical Shift Perturbation (CSP) plots illustrating the interaction of DMSO and NVS-CECR2–1 with the CECR2 bromodomain. Normalized Chemical shifts (Δ ppm) induced by the addition of 3% DMSO are represented in black, while those induced by NVS-CECR2–1 including 3% DMSO) are shown in orange. Prolines are denoted by an asterisk (*), the conserved asparagine N514 by an arrow, and unassigned residues by the # symbol. The dotted line represents the 1 standard deviation cut-off. The secondary structure, comprising four alpha-helices and ZA/BC loop regions of the CECR2-BRD, is delineated above the histogram.

There are currently two known small molecule inhibitors of the CECR2 bromodomain – NVS-CECR2–1 (Structural Genomics Consortium, https://www.thesgc.org/chemical-probes/NVS-CECR2-1) and GNE-886^35^ (Genentech, USA). Of the two, NVS-CECR2–1 has the most potential because it was demonstrated to inhibit the CECR2 - RelA interaction *in vitro*^10^, and CECR2 bromodomain chromatin binding ability^36^. Despite this, the structural details on how and where the inhibitor binds within the CECR2 bromodomain pocket are still missing. Using 2D ^15^N-^1^H HSQC NMR spectroscopy, we defined the binding contacts for the NVS-CECR2–1 inhibitor within the CECR2-BRD (Figure 6). However, since NVS-CECR2–1 is only soluble in DMSO, and since DMSO is itself a competitive bromodomain binding molecule^30, 37^, we first mapped the binding of DMSO to the ^15^N-labeled CECR2-BRD (Figure 6, Supp. Figure S13). As expected, the binding of DMSO resulted in significant (> 1 Δppm) shifts in residues within the ZA loop and BC loop region, including the conserved asparagine (N514). The binding pocket of DMSO is similar to the acetylated ligands – H3, H4, and RelA. Next, we assessed the binding of the inhibitor NVS-CECR2–1 (dissolved in DMSO) to the ^15^N-labeled CECR2-BRD (Figure 6, Supp. Figure S13). Addition of the inhibitor caused CSPs in residues within the expected DMSO binding pocket as mentioned above, however, we also observed CSPs in some residues within αA, αB and αC helices. Overall, our data indicates that DMSO competes with NVS-CECR2–1 for binding to the CECR-BRD, posing challenges in interpreting NVS-CECR2–1 interactions. However, the inhibitor binding pocket aligns with acetylated ligands such as histones H3, H4 and RelA, hinting NVS-CECR2–1 may target a related area. Further structural analyses are essential to fully understand the inhibitor NVS-CECR2–1’s binding mechanism.

### The CECR2 bromodomain prefers binding to multi-acetylated H3 and H4 within the context of the nucleosome

**Figure 7.**
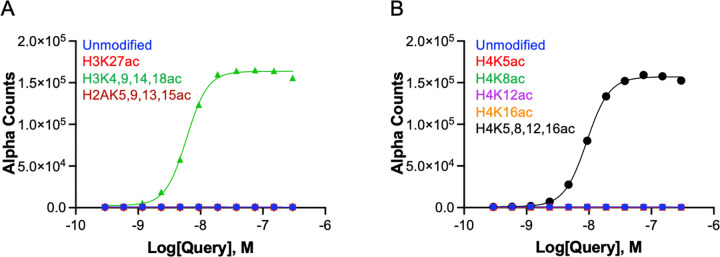
CECR2 bromodomain robustly binds multi-acetylated H3 and H4 nucleosomes. Using the dCypher assay, CECR2 was evaluated against nucleosomes; X-axis represents the concentration of CECR2, while the Y-axis measures the Alpha Counts indicating binding strength. The key identifies each nucleosome. A) CECR2 was assessed against H3K27ac and multi-acetyl H2A and H3 nucleosomes showing strong binding only to H3Tetra^ac^. B) CECR2 interactions to single and multi-acetyl H4 nucleosomes revealed binding only H4Tetra^ac^. All values shown are the averages ± standard deviation (n = 2).

To further elucidate the physiological relevance of CECR2-BRD’s interactions with acetylated histones, we evaluated its binding to PTM-defined nucleosomes. CECR2-BRD demonstrated a clear binding preference for H3Tetra ^ac^ (EC_50_^rel^: 6.0 nM) and H4Tetra^ac^ (EC_50_^rel^: 9.2 nM) nucleosomes (Figure 7, Supp. Figure S14). Notably, no binding was observed to any singly acetylated target. This indicates a potential critical role for multiple acetylation modifications (i.e. weakened electrostatic interactions between DNA and histones) in facilitating CECR-BRD interactions within the nucleosomal context.

## DISCUSSION

This study provides insight on the bromodomain of CECR2, a subunit within ATP-dependent chromatin remodeling complexes including SMARCA5^1, 2^. The bromodomain mediates the selective recognition of acetylated lysine residues in regulating chromatin accessibility. These findings provide an understanding of bromodomain specificity in the context of its described roles in regulating both transcription and replication^38^.

In this study, using a high-throughput and combinatorial array of post-translationally modified histone ligands we found that the CECR2-BRD has a higher affinity towards shorter chain acyl modifications on histones H3 and H4, such as H3K9ac/pr/bu >> H3K9bhb/su/vl/hxo (Figure 1 & 2). These observations correspond well with previous acetylated histone peptide array and pulldown results^15, 30^. The differential effect of adjacent PTMs on the recognition of acetylation marks is worth noting. Methylation was observed to be well-tolerated (Figure 1F), whereas phosphorylation adjacent to acetyllysine residues hindered recognition (Figure 1G). Higher tolerance towards methylation may indicate the ability of the CECR2-BRD to differentiate between various histone states depending on the PTMs nearby. In contrast, phosphorylation potentially serves as a regulatory switch for when CECR2 is recruited. For example, asymmetric segregation of distinct pools of old and new H3 are distinguished by phosphorylation at H3T3 in drosophila germline stem cells, and this tight regulation is integral for proper sister chromatids in prophase^39^. In addition, histone H3S10ph is a conserved PTM involved in chromatin compaction, distinctly associated with dividing cells in metaphase in most organisms^40^. Its role in chromatin compaction, is in part, the result of blocking Heterochromatin Protein 1 from associating with chromosomes during mitosis. Similar to H3T3 phosphorylation in drosophila, H3S10ph is involved with distinguishing “old” vs “new” H3, with this mark being associated with preexisting H3 during early stages of mitosis, while this PTM starts to appear on new H3 as mitosis progresses^41^. H3S10ph has been shown to localize to specific R loops, which are structures composed of newly synthesized RNA, as well as with the template DNA strand and non-template single stranded DNA. R loops are canonically involved in regulating gene transcription, and these structures are also linked with genomic instability^42^. In yeast cells, DNA damage can result from R loop formation. However, mutations in histone H3 at serine 10 that prevented histone phosphorylation were protected from accumulating DNA damage^42^. Experiments by Sharma et. al. suggest that H3S10ph inhibits DNA damage repair machinery. They found inhibition of the primary phosphatases that remove phosphates from H3S10 led to impaired repair of damaged DNA and thus, cell death^43^. Our results indicate that phosphorylation disrupts the interaction between the CECR2 bromodomain and acetylated histone H3 ligands. Phosphorylation of histone H3 promotes chromatin compaction and cell cycle progression, and creates a chromatin state that is less accessible by transcription factors and chromatin binding/remodeling proteins such as CECR2^44^. Additionally, reduced binding between the CECR2 bromodomain and phosphorylated histone H3 may be supported by physiological phenomenon beyond the mechanism of cell cycle progression. Lee et al. utilized siRNA knockdown experiments to demonstrate that CECR2 plays a role in the DNA damage response^5^, while Garcia-Pichardo et al. and Sharma et al. found that phosphorylation of histone H3 leads to impaired DNA repair^42, 43^. Thus, the correlation between H3 phosphorylation and genomic instability due to increased susceptibility to DNA damage could be associated with the impaired binding of the CECR2 bromodomain to H3 in the presence of phosphorylation. The varying impact adjacent methyl and phosphoryl groups have on recognition of acetylation marks suggests a complex mechanism to fine-tune processes that CECR2 regulates, such as gene expression and DNA repair. Furthermore, the identity of residues adjacent to the acetylated lysine also influences bromodomain binding affinity as demonstrated by the preference of CECR2-BRD for H3K14ac, which is surrounded by small non-polar residues. Similarly, oncogenic mutations in histone proteins are known to disrupt bromodomain binding^45^. Similarly, introducing polar amino acids, such as serine and aspartic acid, adjacent to the acetyllysine residue, abolished acetylated histone ligand binding by the ATAD2 and ATAD2B bromodomains^46^.

Notably, acetylation of histone H3 is associated with neural differentiation^47, 48^. Considering that CECR2 itself plays a critical role in the neurulation process^1^, it is reasonable to assume that the CECR2-BRD would be recruited through multiply acetylated histone H3. This aligns well with both our ITC data (Table 1) and the nucleosome assay (Figure 7) that showed a high affinity of CECR2-BRD for histone and nucleosome ligands containing multi-acetylated histone H3 and H4. Hyperacetylation of histone H3 and histone H4 is associated with euchromatic regions where genes are actively expressed^49^. For histone H4, we observe that an increase in the number of acetylated lysine residues corresponds to an increase in binding affinity (Table 1). We cannot conclude that histone H3 binding has the same trend as only mono- and tetra-acetylated ligands were used in ITC, although the peptide array data show high alpha counts between CECR2 and di-acetylated lysines on histone H3, which is indicative of an interaction. For both histone H3 and H4, the tightest binding was exhibited between CECR2-BRD and the tetra-acetylated ligands, supporting the trend that multiple acetylation modifications on histone tails increases the binding affinity for bromodomain-containing proteins^46, 50, 51^. It is worth noting that some acetyllysine marks contribute more to increasing the binding affinity than others^52^. This is also seen in our ITC data for the CECR2-BRD with acetylated histone H4 ligands. For example, the CECR2-BRD has a nearly identical affinity for di-acetylated H4K5acK8ac (*K*_*D*_ = 15.5 ± 1.7 μM) and mono-acetylated H4K8ac (*K*_*D*_ = 15.4 ± 1.9 μM), while the affinity for mono-acetylated H4K5ac is significantly lower (*K*_*D*_ = 63.8 ± 3.8 μM). Acetylation on residue K8 on histone H4 has a larger contribution to an increased binding affinity. This is supported by ITC data tri-acetylated histone H4 ligands where the CECR2-BRD affinity for H4K5acK12acK16ac (*K*_*D*_ = 21.1 ± 1.3 μM) is lower than its affinity for H4K8acK12acK16ac (*K*_*D*_ = 19.9 ± 1.2 μM). We also found that the CECR2-BRD exhibits a stronger binding affinity for histone H4 ligands compared to histone H3 ligands. These results provide new insights into the role of CECR2-BRD in nucleosome interactions and its function in regulation. According to relevant literature, histone H3 acetylation is directly associated with transcriptional activation through interaction with transcription factors, while histone H4 acetylation is indirectly associated with transcriptional activation by impacting the structure and stability of the nucleosome^53, 54^. Histone H4 acetylation influences the accessibility to DNA wrapped around the nucleosome, while histone H3 acetylation potentially affects nucleosome-nucleosome interactions^53, 54^. Our ITC and the nucleosome binding data demonstrate that CECR2-BRD binds tightly to tetra-acetylated histone H3 and H4 ligands and nucleosomes. This strengthens the idea that CECR2 is a ‘key epigenetic regulator’^4^, as it interacts with modifications associated with transcriptional activation.

Interestingly, in contrast to our peptide array results, the CECR-BRD did not bind to any nucleosomes containing singly acetylated histones or tetra-acetylated H2AK5ac9ac13ac15ac histones (Figure 7). This indicates the critical role of acetylation in increasing histone tail accessibility, facilitating specific chromatin interactions. These findings highlight the importance of multi-acetylation for the specificity and recognition of histone tail by CECR2-BRD in the context of the nucleosome.

Similar to acetylation, CECR2-BRD exhibited a preference for propionyl and butyryl modifications on H3 and H4 peptides (Figure 2), suggesting a broader recognition of short-chain acyl groups. In contrast, crotonylation markedly diminished binding, while bulkier acyl groups such as beta-hydroxybutyryl and hexanoyl completely eliminated interaction with CECR2-BRD. Our results are consistent with the previous findings^30^ that confirmed CECR2-BRD recognized butyryl and propionyl modifications on histone H4 but failed to bind crotonyl H4. Our data supports the view that bromodomains can perform nuanced regulatory processes through their selective recognition of multivalent histones.

Our results from AUC experiments indicate that CECR2 BRDs binding of histone ligands occurs in a monomeric fashion, and the majority of protein is monomeric in solution. Our NMR study focused on elucidating the molecular mechanisms that drive the specificity in recognizing different acetylation marks by the CECR2-BRD. A detailed comparison of the CSPs upon addition of acetylated histone H3 and H4 peptides reveals they are coordinated similarly within the CECR2 BRD binding pocket (Figure 5). The CECR2-BRD residues most perturbed upon histone ligand addition are residues located in the ‘WPF’ shelf region, the BC loop, and the ZA loop containing the conserved N514. The perturbation of these residues is consistent with the literature and indicates the CECR2 BRD uses the canonical mode of acetyllysine recognition^55^. It was revealed that CECR2-BRD recognizes acetylated histone H3 independently of the number of acetylation modifications, while more intense CSPs were observed for multi-acetylated histone H4 compared to the mono-acetylated ligand. When D464 was mutated to Ala, a modest decrease in binding was observed, as shown by our ITC data. On the contrary, N514 mutated to Ala significantly reduced binding, indicative of the conserved residue’s importance. Within the tetra-acetylated histone ligands examined by ITC, the N514A mutation had a more pronounced effect on the recognition of tetra-acetylated histone H4 than tetra-acetylated histone H3 compared to wild-type CECR2 (Figure 5). Overall, our data suggests that interactions between CECR2-BRD and the mono-acetylated histone ligands is mediated largely by the conserved residue N514, possibly by creating stabilizing contacts with these ligands thereby impacting their binding affinity. However, charged residues within the CECR2-BRD pocket such as D464 may play some role in modulating the histone interaction which may not directly impact their binding affinity.

We also found that the bromodomain of CECR2 interacts with acetylated RelA in a distinct mode differing from interaction with histone ligands. Acetylation of the transcription factor p65 (RelA), a member of the NF-κB family, modulates inflammatory responses and drives many of the pathophysiological functions of NF-κB^8, 9, 32, 33^. Specifically, acetylation of Lys310 of RelA is required to fully activate the transcriptional functions of NF-κB^9, 34^. Recently, CECR2 was identified as a critical epigenetic regulator that interacts with acetylated RelA, increasing the expression of NF-κB target pro-inflammatory genes and driving breast cancer metastasis^10^. Overall, the CECR2-BRD coordinates the RelAK310ac ligand similarly to the histone H3 or H4 interactions. However, there are some notable differences, particularly with residues within the ZA and BC loop regions of the CECR2-BRD, where these unstructured loop regions display larger structural rearrangements upon RelAK310ac ligand binding (Figure 5). Specifically, A468, N470, and Y472 are more perturbed upon CECR2 binding to acetylated RelA, whereas CSPs in these residues are weaker in the presence of acetylated histone H3 and H4 ligands. Conversely, binding of acetylated histone ligands induces stronger CSPs in residues in the canonical bromodomain ligand binding pocket. These differences indicate a distinct binding mode is used by CECR2 to selectivity recognize both histone and non-histone ligands. However, the conserved asparagine (N514) plays a key role in coordinating both acetylated RelA and acetylated histone ligands, since mutation of N514 to Ala resulted in abolishing RelAK310ac binding (Table 3). Other bromodomain-containing proteins are known to bind acetylated lysines on non-histone proteins^56^. The sequence motifs found in non-histone ligands are not as conserved as they are in histone ligands, and the CECR2-BRD must adapt to accommodate recognition of acetylated RelA through additional contacts that stabilize the interaction. Additionally, Huang et al. found the bromodomain of Brd4 also binds to acetylated lysine 310 on RelA, which leads to enhanced transcriptional activation of NF-kB^57^. This result suggests overlapping roles between Brd4 and CECR2, however they did not examine the specific binding mechanism, thus it is unclear if Brd4 and CECR2 utilize a conserved binding mode to interact with RelA.

Previous studies have shown that the CECR2 inhibitor, NVS-CECR2–1, can inhibit interactions between CECR2 and RelA *in vitro* as well as inhibit the chromatin binding abilities of the CECR2-BRD^36^. Our NMR data indicate that the CECR2-BRD uses a distinct binding mode to distinguish between acetylated histones and acetylated RelA as the chemical shift perturbation patterns for the histone vs non-histone ligands show significant differences (Figure 5). However, the predominant residues necessary for these interactions are localized to the ZA and BC loops. Interestingly, while the downstream effects of NVS-CECR2–1 correspond to blocking interactions between CECR2 and chromatin as well blocking CECR2 with RelA, our data suggests the NVS-CECR2–1 inhibitor binds to residues that are distinct from those involved in canonical bromodomain recognition of acetyllysine on histones and RelA. Our 2D HSQC NMR data shown in Figure 6 suggests that NVS-CECR2–1 binds to the αA, αB, and αC secondary structures of the CECR2-BRD. Interestingly, Crawford et.al found that the pyridone carbonyl and pyrrole of another CECR2-BRD inhibitor, GNE-886, forms hydrogen bonds with the N514, a highly conserved residue within the BC loop of the CECR2-BRD (PDBID: 3NXB), while the core pyrrolopyridone of the inhibitor forms π-stacking interactions with the gatekeeper Y520^35^. The piperidine participates in hydrophobic interactions with W457 of the WPF motif and Y467 of the αZ helix. There are additional hydrophobic interactions between F459, P458 and Y520 of the WPF shelf with the allyl group of GNE-866 at the 4-carboxamide, while the carbonyl group of the N-methylpiperidine amide interacts with ZA loop at D464 via hydrogen bonding. When we assessed the binding of NVS-CECR2–1 (dissolved in DMSO) to ^15^N-labeled CECR2-BRD via 2D HSQC NMR (Figure 6) we observed no shifts overlapping with residues involved in coordinating GNE-886. This could be the result of the structural differences between the two inhibitors, that result in different binding modes that are dependent on the specific ligand. Coordination of acetylated histone peptides and acetylated RelA appear to happen through a slightly different subset of amino acids, but the residues in the ZA and BC loops that are involved in binding to tetra-acetylated histone H3 and H4 ligands and acetylated RelA are somewhat conserved in the coordination of GNE-866. These include residues F459, N514, and D464. Potentially the carbonyl and the allyl-methyl groups of the pyridine in GNE-866 serve as the acetyl-lysine (Kac) mimetic^35^, which are not present on NVS-CECR2–1, which could explain the alternate interaction mode located outside the canonical acetyllysine binding pocket.

Our results demonstrate the histone-binding preferences of the CECR2-BRD and identify critical residues within its binding pocket that drive the distinct binding modalities observed between histone and non-histone ligands. These results support a model where the CECR2-BRD plays an important role in facilitating the interaction of CECR2 with chromatin where it functions as an epigenetic regulator of gene expression. However, characterizing the BRD of CECR2 in isolation, rather than the full-length protein, introduces some limitations to the conclusions of our studies. The regulation of gene expression can be extremely nuanced and context-dependent, and while our results inform initial interaction mechanisms between CECR2 and other regulatory proteins, these interactions may differ in the context of the full-length protein. Furthermore, the use of modified histone ligands in our studies, while this is an accepted technique for structural and functional studies, it has additional limitations. These ligands cannot capture the entirety of that interactions (steric, ionic, hydrophobic, etc.) that occur within the context of the nucleosome. Despite these limitations, our results establish a foundation for further in-depth investigations into the role of CECR2 as a regulator of gene expression and inflammation.

The functional implications of non-histone protein acetylation have been explored in various cellular processes^58^. Non-histone protein acetylation regulates p53-mediated apoptosis^59^, NF-κB signaling^7^, and metabolic reprogramming in cancer cells^58^. In breast cancer, dysregulation of NF-κB signaling drives metastasis^60^. CECR2 was recently recognized as a ‘top epigenetic regulator’ that upregulates NF-κB inflammatory and pro-metastatic genes, emphasizing the therapeutic potential of targeting this bromodomain-containing protein in cancer treatment. Understanding the intricate differences in bromodomain recognition of histone versus non-histone acetylation marks is critical for elucidating the role of these epigenetic mechanisms in the initiation and progression of various diseases. These findings provide strong support for further investigation into CECR2-BRD as a therapeutic target and continued exploration of bromodomain roles in disease contexts.

## CONCLUSION

In conclusion, the research presented in this study sheds light on the recognition and specificity determinants of the CECR2-BRD towards acetylation on histone versus non-histone proteins. Our findings demonstrate that the CECR2-BRD exhibits an ability to recognize a broad range of acylated lysine modifications, accommodating diverse combinations of PTMs on histone H3 and H4 tails. Through NMR titration experiments, we found that the CECR2-BRD uses a distinct binding mode to interact with acetylated histones in comparison with an acetylated RelA peptide. Moreover, site-directed mutagenesis experiments identified key residues within the ZA-BC loop that govern the selective recognition of histone versus non-histone substrates. Overall, this study enhances our knowledge of CECR2-BRD function in coordinating specific PTMs, laying the groundwork for further investigations into the molecular mechanisms underlying the bromodomain-mediated epigenetic regulation and its potential implications in disease pathogenesis and therapeutic development.

## Supplementary Material

Supplement 1

Supplement 2

## Figures and Tables

**Table 1:** CECR2 bromodomain binding to acetylated H3 and H4 histone ligands measured by ITC. The binding affinities are represented as K_D_ (μM), and N denotes stoichiometry. All peptides are 1 to 24 residues long.

Histone Ligand	Amino Acid sequence	*K*_D_ (μM)	N
**Histone H3**			
H3 unmodified	ARTKQTARKSTGGKAPRKQLATKA	No binding	-
H3K4ac	ART**Kac**QTARKSTGGKAPRKQLATKA	114.3 ± 7.2	1.0 ± 0.0
H3K9ac	ARTKQTAR**Kac**STGGKAPRKQLATKA	83.0 ± 1.3	1.0 ± 0.1
H3K14ac	ARTKQTARKSTGG**Kac**APRKQ	34.2 ± 5.0	0.9 ± 0.1
H3K18ac	ARTKQTARKSTGGKAPR**Kac**QLATKA	60.7 ± 3.0	0.9 ± 0.1
H3K4acK9acK14acK18ac	ART**Kac**QTAR**Kac**STGG**Kac**APR**Kac**QLATKA	28.0 ± 1.7	1.1 ± 0.1
**Histone H4**			
H4 unmodified	SGRGKGGKGLGKGGAKRHRKVLRD	No Binding	-
H4K5ac	SGRG**Kac**GGKGLGKGGAKRHRKVLRD	63.8 ± 3.6	1.0 ± 0.0
H4K8ac	SGRGKGG**Kac**GLGKGGAKRHRKVLRD	15.4 ± 1.9	1.0 ± 0.0
H4K12ac	SGRGKGGKGLG**Kac**GGAKRHRKVLRD	92.1 ± 6.7	1.0 ± 0.0
H4K16ac	SGRGKGGKGLG**Kac**GGAKRHRKVLRD	41.1 ± 3.9	1.0 ± 0.1
H4K5acK8ac	SGRG**Kac**GG**Kac**GLGKGGAKRHRKVLRD	15.5 ± 1.7	1.0 ± 0.0
H4K12acK16ac	SGRGKGGKGLG**Kac**GGA**Kac**RHRKVLRD	23.0 ± 0.9	1.1 ± 0.0
H4K5acK12acK16ac	SGRG**Kac**GGKGLG**Kac**GGA**Kac**RHRKVLRD	21.1 ± 1.3	0.8 ± 0.0
H4K8acK12acK16ac	SGRGKGGKGLG**Kac**GGA**Kac**RHR**Kac**VLRD	19.9 ± 1.2	1.0 ± 0.0
H4K5acK8acK12acK16ac	SGRG**Kac**GG**Kac**GLG**Kac**GGA**Kac**RHRKVLRD	1.6 ± 0.2	1.0 ± 0.0

**Table 2:** CECR2 bromodomain mutants binding to acetylated histone H3 and H4 ligands measured by ITC. The binding affinities are represented as *K*_D_ (μM), and N denotes stoichiometry. All peptides include residues 1–24.

Histone Ligands	Protein	Dissociation Constant *K*_D_ (μM)	N
H3K14ac (1–24)	CECR2 WT	34.2 ± 5.1	0.9 ± 0.1
	CECR2 D464A	74.4 ± 3.0	1.1 ± 0.2
	CECR2 N514A	560.5 ± 54.4	1.0 ± 0.0

H3K4acK9acK14acK18ac (1–24)	CECR2 WT	28.0 ± 1.7	1.1 ± 0.1
	CECR2 D464A	63.3 ± 2.3	1.0 ± 0.0
	CECR2 N514A	28.4 ± 3.4	1.1 ± 0.3

H4K8ac (1–24)	CECR2 WT	15.4 ± 1.9	1.0 ± 0.0
	CECR2 D464A	239.0 ± 18.5	1.3 ± 0.4
	CECR2 N514A	518.7 ± 84.6	1.0 ± 0.0

H4K5acK8acK12acK16ac (1–24)	CECR2 WT	1.5 ± 0.2	1.0 ± 0.0
	CECR2 D464A	9.7 ± 0.8	1.0 ± 0.0
	CECR2 N514A	37.4 ± 5.4	0.9 ± 0.0

**Table 3:** CECR2 bromodomain mutants binding to RelAK310ac measured by ITC. The binding affinities are represented as K_D_ (μM), and N denotes stoichiometry. The peptide includes RelA residues 305–315.

Ligand	Amino Acid Sequence	Protein	Dissociation Constant *K*_D_ (μM)	N
RelAK310 (305–315)	TYETFKSIMKK-NH2	WT	No Binding	-
RelAK310ac (305–315)	TYETF**Kac**SIMKK-NH2	WT	112.3 ± 4.2	1.0 ± 0.0
		D464A	490.3 ± 67.0	1.0 ± 0.0
		N514A	No Binding	-

## HIGHLIGHTS:

- The CECR2 bromodomain recognizes a range of combinatorial PTMs on the histone H3 and H4 N-terminal tails.
- The CECR2 bromodomain binds to an acetylated RelA ligand with micromolar affinity.
- NMR perturbation studies delineate the distinct binding modes driving CECR2-BRD recognition of histone versus non-histone ligands.
- Site-directed mutagenesis reveals the specificity determinants of CECR2-BRD ligand binding.
- The bromodomain of CECR2 exhibits a strong interaction with multi-acetylated nucleosomes.
