# Supplementary material for: The CECR2 bromodomain displays distinct binding modes to select for acetylated histone proteins versus non-histone ligands
Source: bioRxiv. 2024 Dec 11:2024.12.09.627393. Preprint. [Version 2] doi: 10.1101/2024.12.09.627393 (PMC11661176; doi:10.1101/2024.12.09.627393)
Supplement: Supplement 1 [file media-1.pdf]

SUPPLEMENTAL INFORMATION

The CECR2 bromodomain displays distinct binding modes to select for acetylated histone proteins versus non-histone ligands.

Margaret Phillips[1], Elizabeth D. Cook[1], Matthew R. Marunde[2], Marco Tonelli[3], Laiba Khan[2], Amy Henrickson[4], James M. Lignos[1], Janet L. Stein[5,7], Gary S. Stein[5,7], Seth Frietze[6,7], Borries Demeler[4], and Karen C. Glass[1,7*]

[1]Department of Pharmacology, Larner College of Medicine, University of Vermont, Burlington, VT, 05405, USA

[2]EpiCypher Inc., Durham, North Carolina 27709, USA

[3]National Magnetic Resonance Facility at Madison and Department of Biochemistry, University of Wisconsin-Madison, Madison, WI, 53706, USA

[4]Department of Chemistry and Biochemistry, University of Lethbridge, Lethbridge, AB T1K 3M4, Canada

[5]Department of Biochemistry, Larner College of Medicine, University of Vermont, Burlington, VT, 05405, USA

[6]Department of Biomedical and Health Sciences, University of Vermont, Burlington, VT 05405, USA.

[7]University of Vermont Cancer Center, Burlington, VT 05405, USA.

* To whom correspondence should be addressed. Tel: +1(802) 656-5760; Fax: +1(802) 656-4523; Email: karen.glass@med.uvm.edu

Running title: Acetyllysine Recognition by the CECR2 Bromodomain

Table of Contents

Supp. Figure S1: dCypher Analysis of the CECR2 bromodomain……….……………..3
Supp. Figure S2: ITC analysis of acetylated histone H3 binding to the CECR2-BRD…..4
Supp. Figure S3: ITC analysis of acetylated histone H4 binding to the CECR2-BRD…..5
Supp. Figure S4: NMR assignment for the CECR2 bromodomain………………….…6
Supp. Figure S5: NMR titration of the CECR2 bromodomain with histone H3K9ac…....7
Supp. Figure S6: NMR titration of the CECR2 bromodomain with histone H3K4acK9acK14acK18ac……………………………………………….………....8
Supp. Figure S7: NMR titration of the CECR2 bromodomain with histone H4K5ac….…9
Supp. Figure S8: NMR titration of the CECR2 bromodomain with histone H4K5acK8acK12acK16ac……………………………………………………………10
Supp. Figure S9: ITC analysis of acetylated ligand binding to the mutant D464A CECR2-BRD..………………………...……………………………………………..11
Supp. Figure S10: ITC analysis of acetylated ligand binding to the mutant N514A CECR2-BRD..……………………………………………………………………………12
Supp. Figure S11: ITC analysis of RelA binding to the CECR2-BRD……………………13
Supp. Figure S12: NMR titration of the CECR2 bromodomain with acetylated RelAK310ac……………………………………………………………………….…...14
Supp. Figure S13: NMR titration of the CECR2 bromodomain with DMSO and NVS-CECR2-1……….………………..15
Supp. Figure S14: Nucleosome-based dCypher assay…………………...………………16

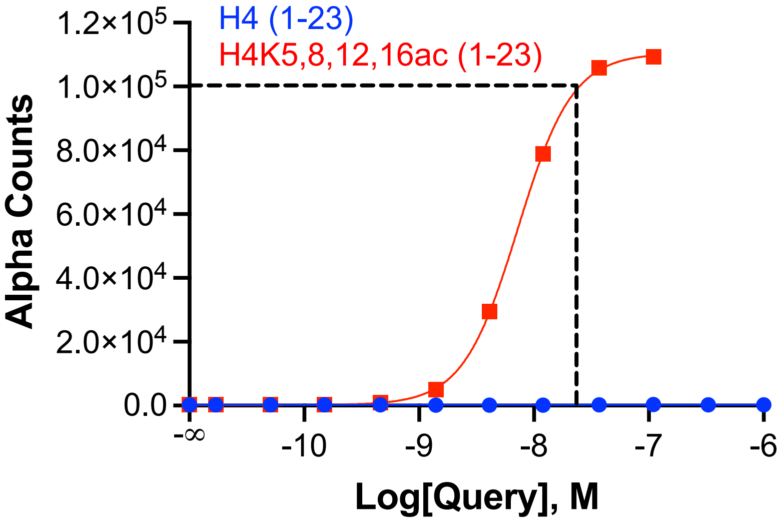

Supp. Figure S1: dCypher Analysis of the CECR2 bromodomain. The dCypher assay was used to titrate GST-CECR2-BRD against unmodified H4 and H4K5ac,8ac,12ac,16ac peptides (res 1-23) to identify the optimal probing concentration (robust signal-to-background) for peptide panel screens. The dashed line represents the concentration of GST-CECR2-BRD (26 nM) chosen for peptide panel screens (Figure 1A-G & Figure 2A-B).

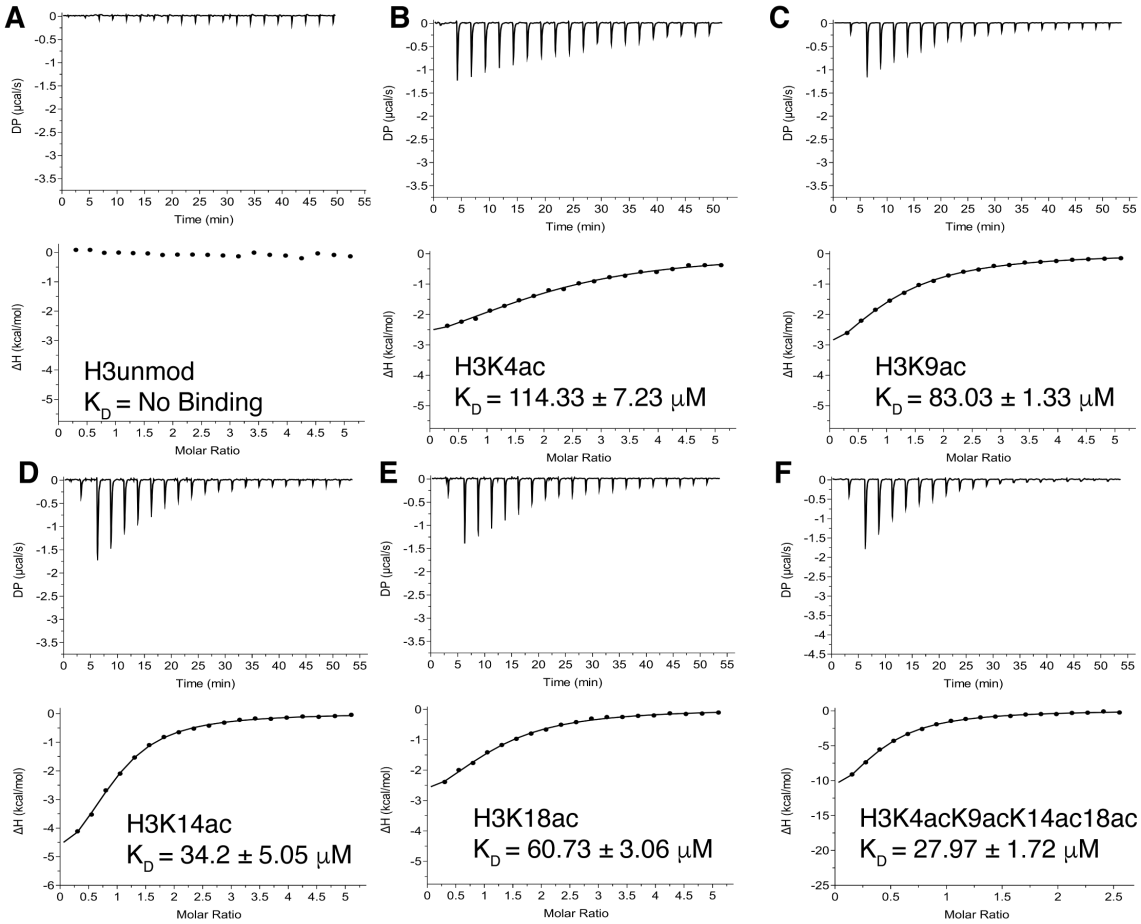

Supp. Figure S2: ITC analyses of the interaction between the CECR2 bromodomain and the acetylated histone H3 peptides. A-F) Exothermic enthalpy plots from ITC experiments depicting the binding interactions between histone H3 ligands (carrying different acetylation marks) and the CECR2 bromodomain. The apparent dissociation constants calculated are indicated for each plot. All peptides are 1-24 amino acids long. Peptide sequences are mentioned in Table 1.

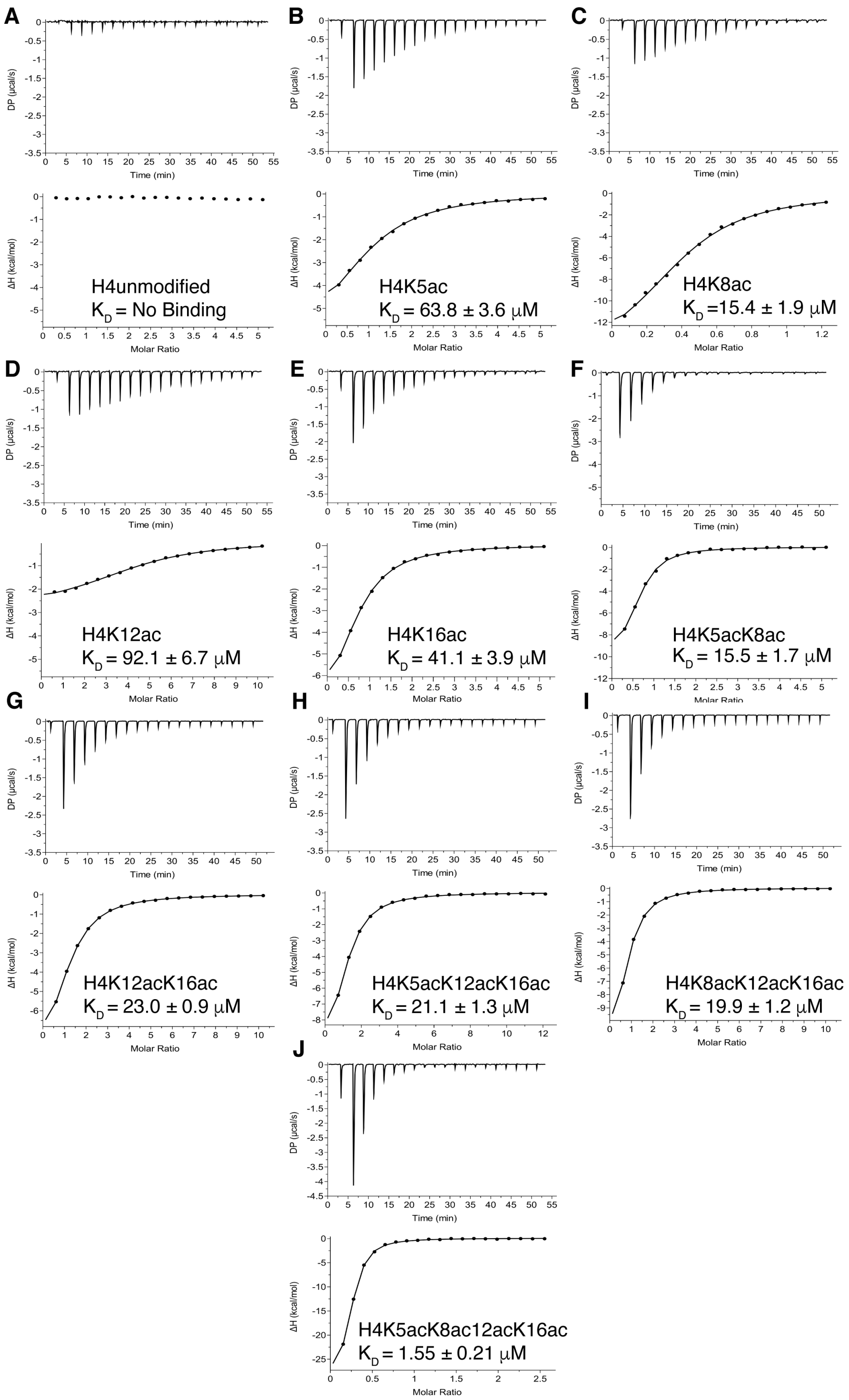

Supp. Figure S3: ITC analyses of the interaction between the CECR2 bromodomain and the acetylated histone H4 peptides. A-J) Exothermic enthalpy plots from ITC experiments depicting the binding interactions between histone H4 ligands (carrying different acetylation marks) and the CECR2 bromodomain. The apparent dissociation constants calculated are indicated for each plot. All peptides are 1-24 amino acids long. Peptide sequences are mentioned in Table 1.

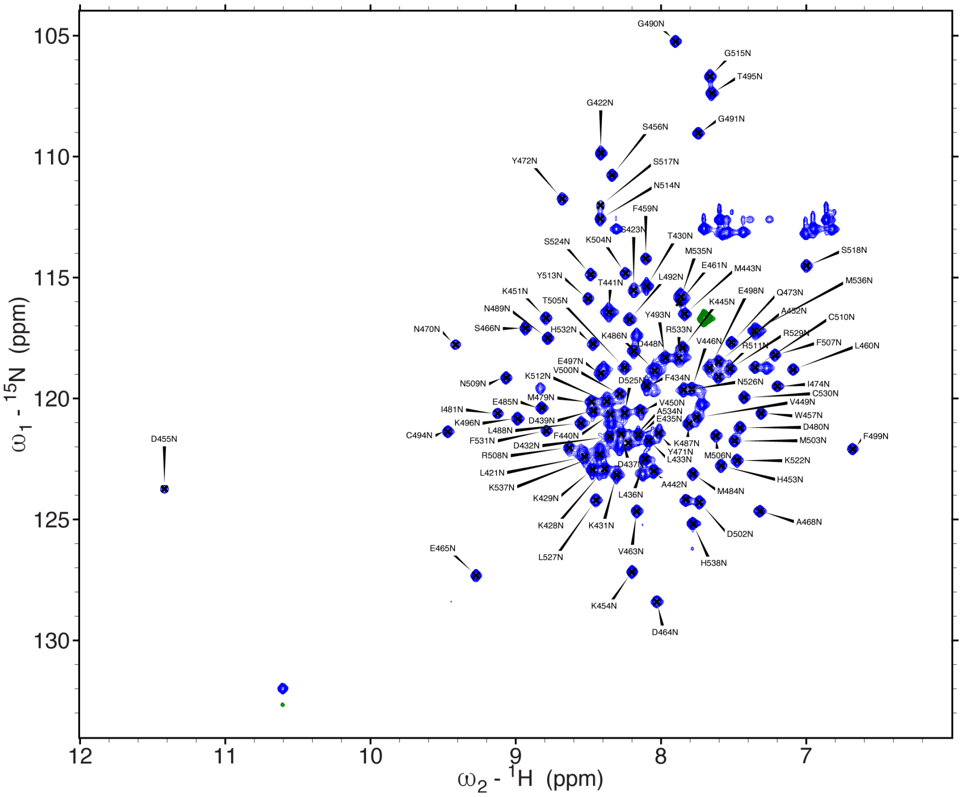

Supp. Figure S4: 2D ^{1}H-^{15}N HSQC Spectrum of the CECR2 Bromodomain with assignment. A total of 106 peaks were observed out of the expected 116, with an assignment rate of approximately 88% (94/116).

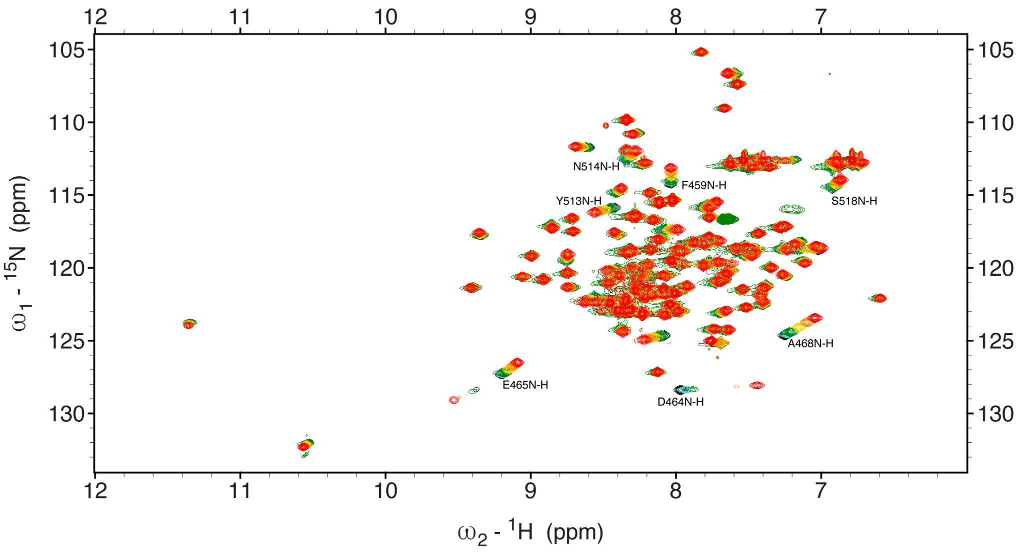

Supp. Figure S5: Interaction of the CECR2 bromodomain with mono-acetylated histone H3K9 ligand. Superimposed 1H-15N HSQC spectra of the CECR2 bromodomain during titration with the H3K9ac (1-24) peptide at 25°C, with color-coded representations indicating increasing molar concentrations of the peptide - black for Apo CECR2 bromodomain, cyan for 1:0.5, green for 1:1, yellow for 1:2.5, orange for 1:5, and red for 1:10 molar ratio of CECR2 and H3K9ac. To avoid overcrowding of the spectrum, only the CECR2 residues undergoing highest chemical shift perturbations (CSP) are labeled.

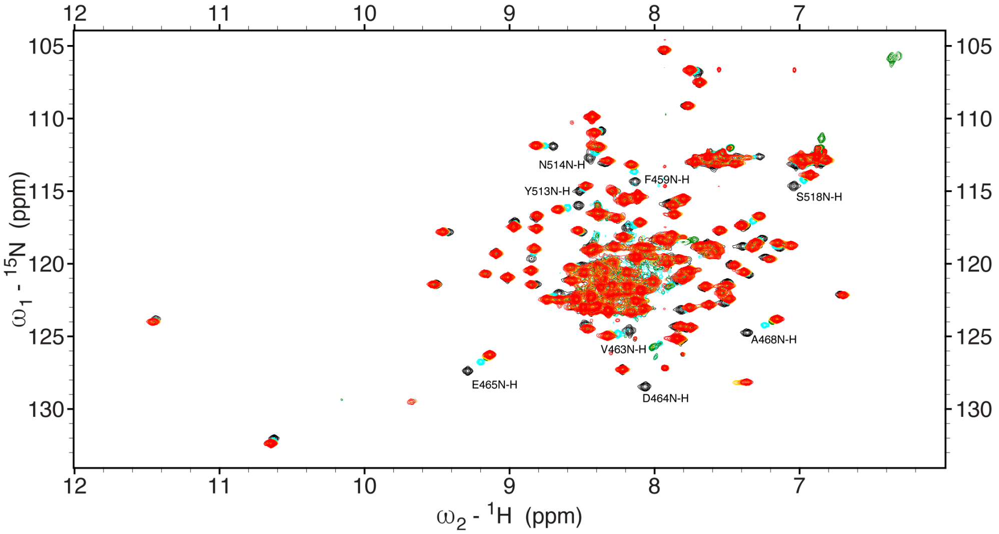

Supp. Figure S6: Interaction of the CECR2 bromodomain with tetra-acetylated histone H3 ligand. Superimposed 1H-15N HSQC spectra of the CECR2 bromodomain during titration with the H3K4acK9acK14acK18ac (1-24) peptide at 25°C, with color-coded representations indicating increasing molar concentrations of the peptide - black for Apo CECR2 bromodomain, cyan for 1:0.5, green for 1:1, yellow for 1:2.5, orange for 1:5, and red for 1:10 molar ratio of CECR2 and peptide. CECR2 residues with maximum CSP are labelled.

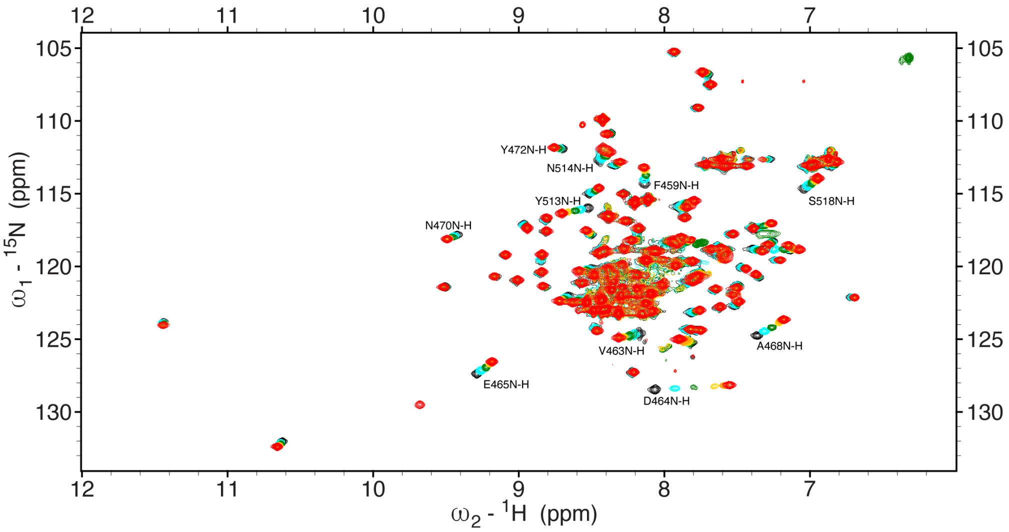

Supp. Figure S7: Interaction of the CECR2 bromodomain with mono-acetylated histone H4K5ac ligand. Superimposed 1H-15N HSQC spectra of the CECR2 bromodomain during titration with the H4K5ac (1-24) peptide at 25°C, with color-coded representations indicating increasing molar concentrations of the peptide - black for Apo CECR2 bromodomain, cyan for 1:0.5, green for 1:1, yellow for 1:2.5, orange for 1:5, and red for 1:10 molar ratio of CECR2 and peptide. CECR2 residues with maximum CSP are labelled.

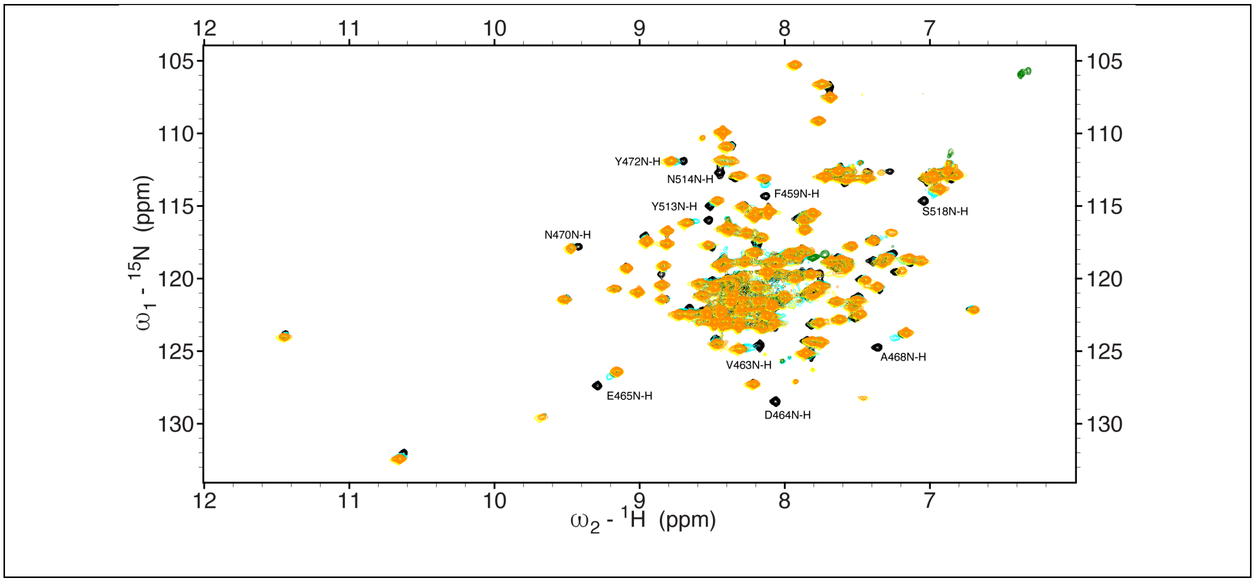

Supp. Figure S8: Interaction of the CECR2 bromodomain with tetra-acetylated histone H4 ligand. Superimposed 1H-15N HSQC spectra of the CECR2 bromodomain during titration with the H4K5acK8acK12acK16ac (1-24) peptide at 25°C, with color-coded representations indicating increasing molar concentrations of the peptide - black for Apo CECR2 bromodomain, cyan for 1:0.5, green for 1:1, yellow for 1:2.5, and orange for 1:5 molar ratio of CECR2 and peptide. CECR2 residues with maximum CSP are labelled.

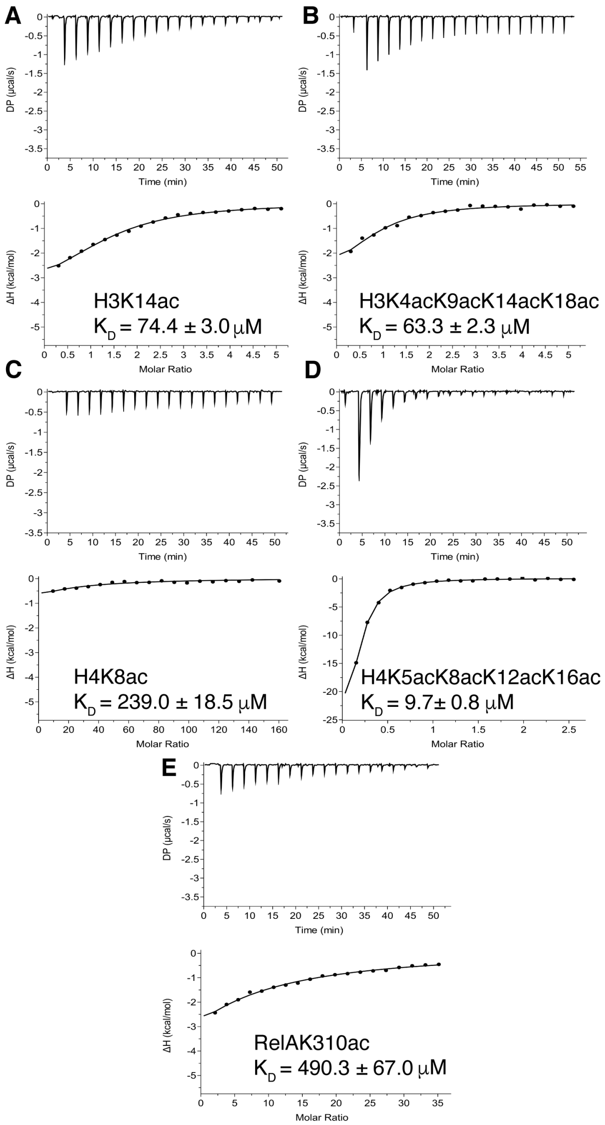

Supp. Figure S9: ITC analyses of the interaction between the D464A mutant of CECR2 bromodomain and the acetylated ligands. A-E) Exothermic enthalpy plots from ITC experiments depicting the binding interactions between acetylated ligands (carrying different acetylation marks) and the mutant CECR2 bromodomain. The apparent dissociation constants calculated are indicated for each plot. All peptides are 1-24 amino acids long except RelAK310 which is 305-315 amino acids long. Peptide sequences are mentioned in Table 1 & 3.

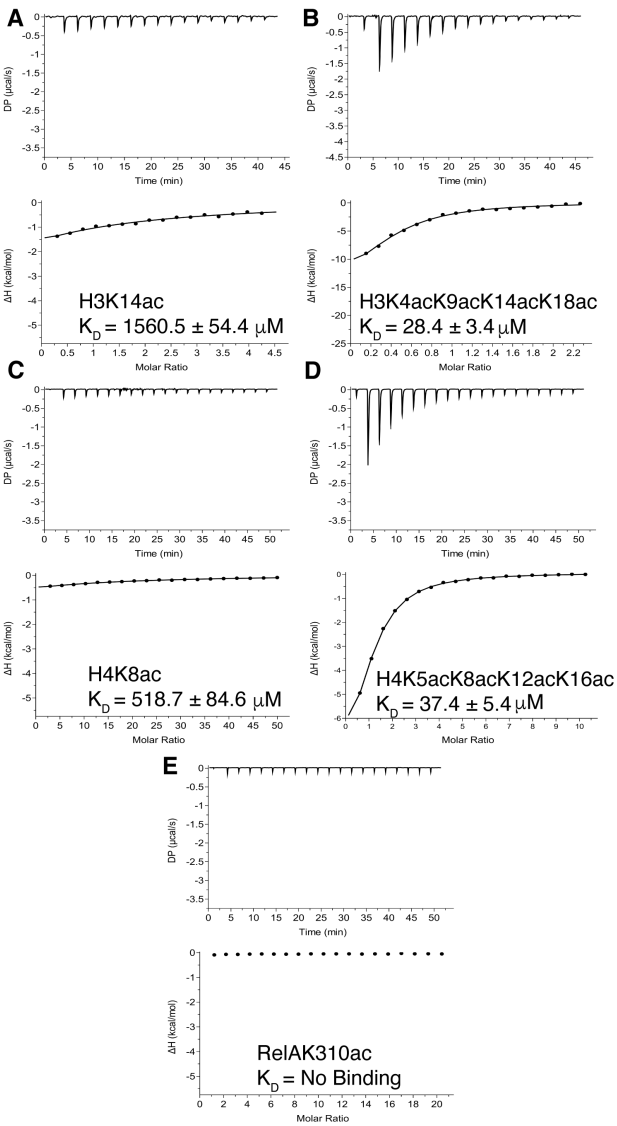

Supp. Figure S10: ITC analyses of the interaction between the N514A mutant of CECR2 bromodomain and the acetylated ligands. A-E) Exothermic enthalpy plots from ITC experiments depicting the binding interactions between acetylated ligands (carrying different acetylation marks) and the mutant CECR2 bromodomain. The apparent dissociation constants calculated are indicated for each plot. All peptides are 1-24 amino acids long except RelAK310 which contains residues 305-315. Peptide sequences are mentioned in Table 1 & 3.

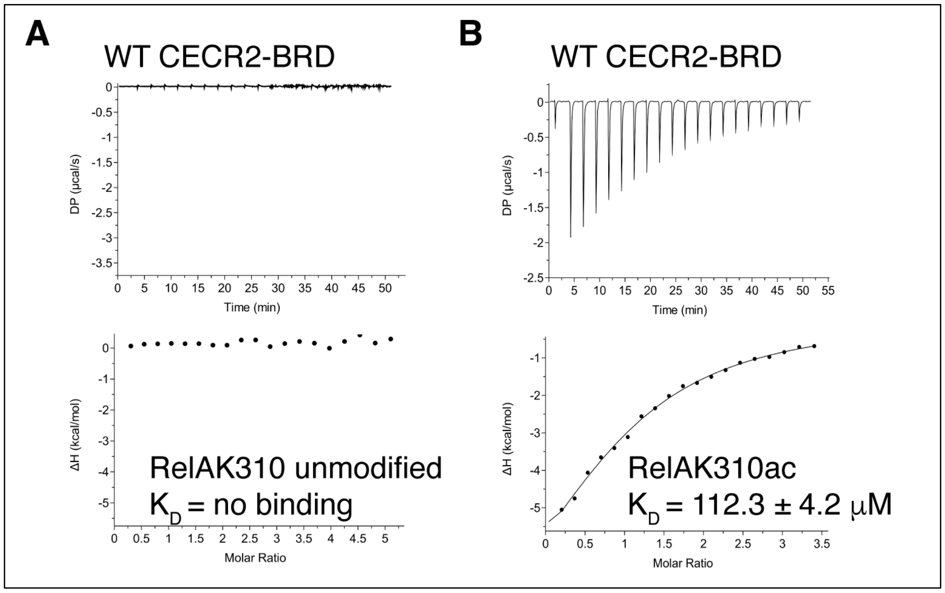

Supp. Figure S11: ITC analyses of the interaction between RelA and the CECR2 bromodomain. A-B) Exothermic enthalpy plots from ITC experiments depicting the binding interactions between RelA ligands (unmodified and acetylated) and the CECR2 bromodomain. The apparent dissociation constants calculated are indicated for each plot. All peptides are 11 amino acids long. Peptide sequences are mentioned in Table 3.

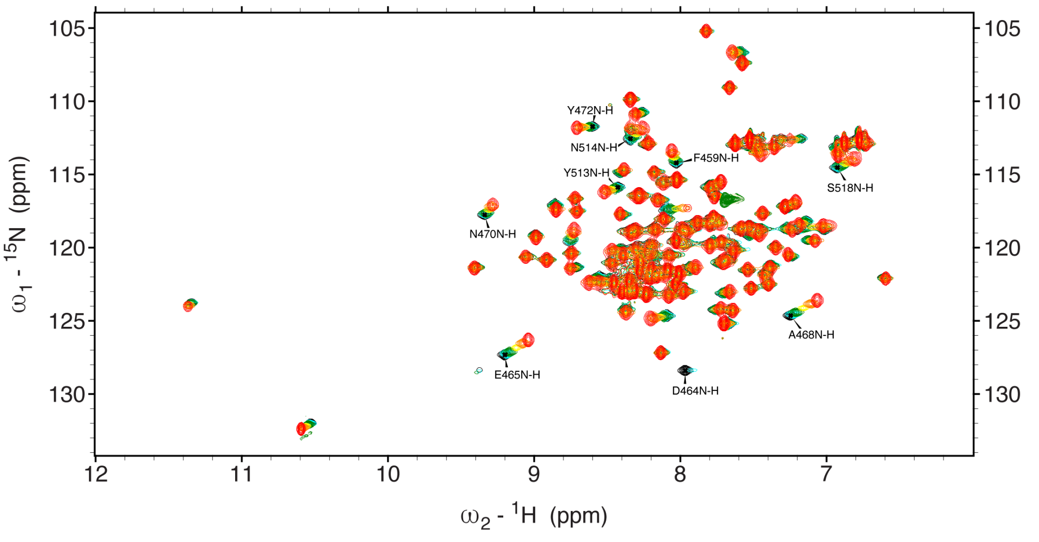

Supp. Figure S12: Interaction of the CECR2 bromodomain with mono-acetylated RelA ligand. Superimposed 1H-15N HSQC spectra of the CECR2 bromodomain during titration with the RelAK310ac peptide at 25°C, with color-coded representations indicating increasing molar concentrations of the peptide - black for Apo CECR2 bromodomain, cyan for 1:0.5, green for 1:1, yellow for 1:2.5, orange for 1:5, and red for 1:10 molar ratio of CECR2 and peptide. CECR2 residues with maximum CSP are labelled.

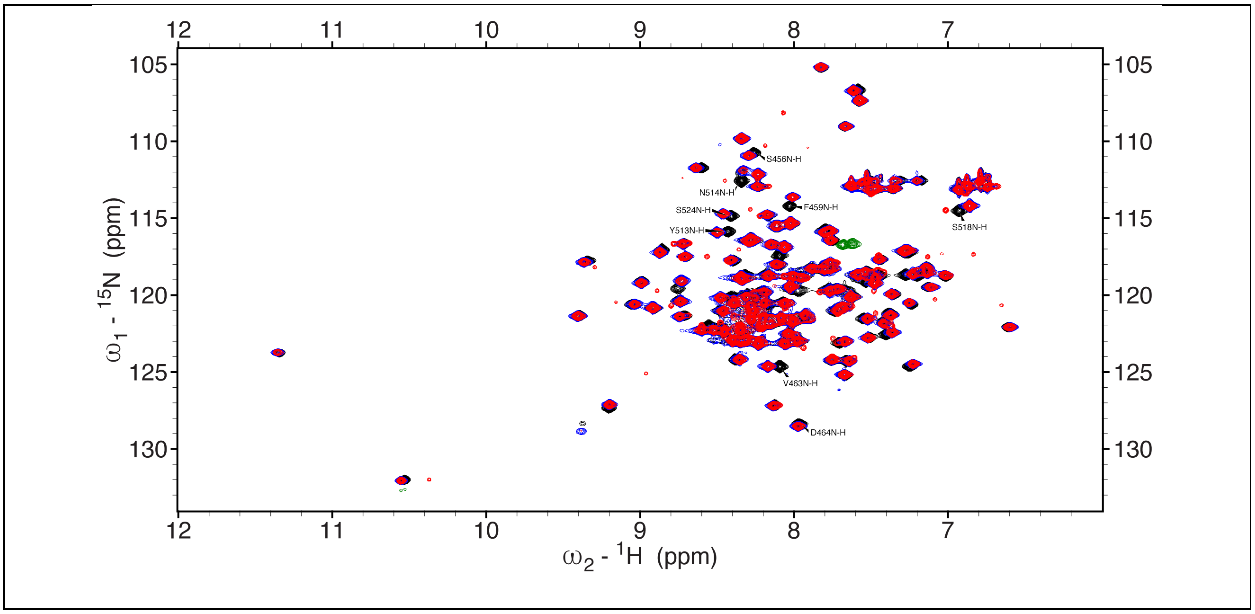

Supp. Figure S13: Interaction of the CECR2 bromodomain with the CECR2 inhibitor (NVS-CECR2-1). A) Overlay of the 2D, ^{1}H-^{15}N HSQC spectra of the CECR2 bromodomain in the absence (black), in the presence of 3% DMSO alone (blue) and upon addition of NVS-CECR2-1 (red) at a 1:1 molar ratio of protein to ligand. All CECR2 residues with maximum CSP are labelled.

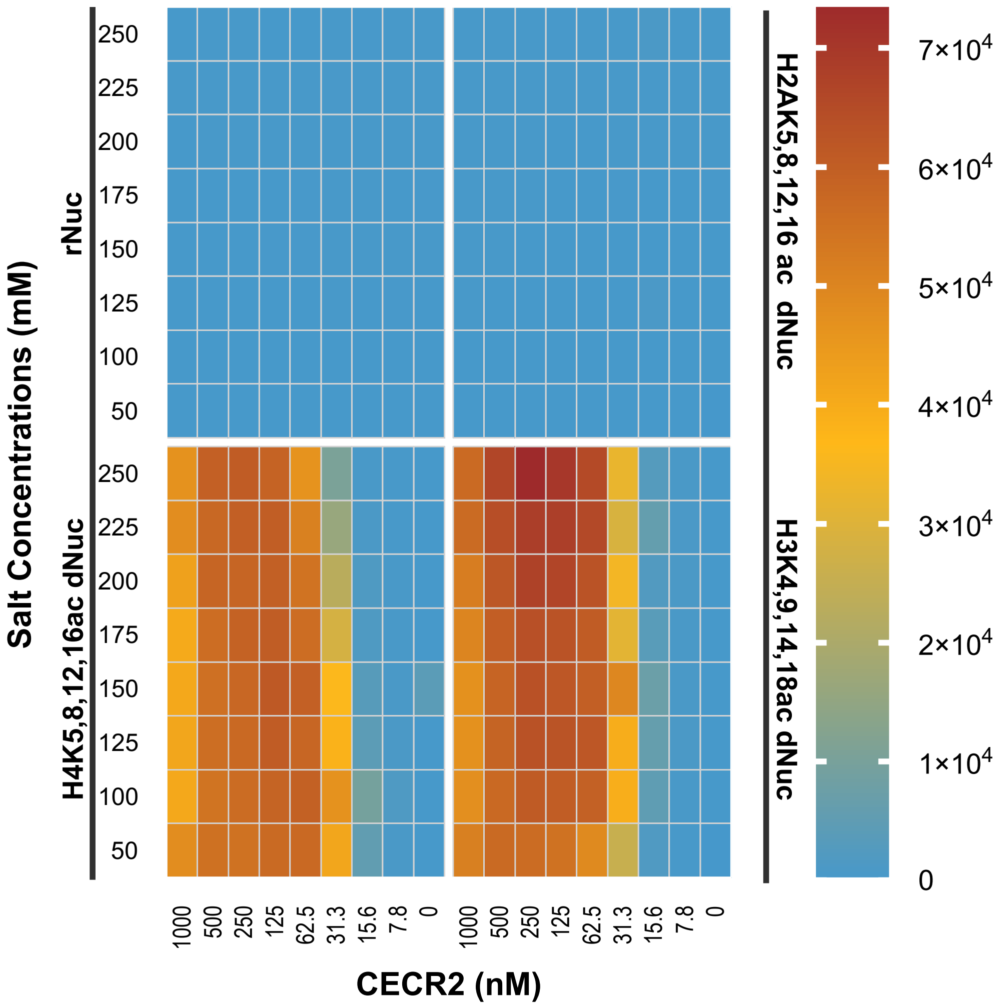

Supp. Figure S14: Nucleosome-based dCypher assay. Using a nucleosome-based dCypher assay both GST-CECR2-BRD and salt condition (NaCl, mM) were titrated to determine the optimal concentration range and buffer condition. The X-axis represents concentration of GST-CECR2-BRD and Y-axis is concentration of NaCl. Alpha counts are represented by color key.
